# MAPK signaling is necessary for neurogenesis in *Nematostella vectensis*

**DOI:** 10.1186/s12915-016-0282-1

**Published:** 2016-08-01

**Authors:** Michael J. Layden, Hereroa Johnston, Aldine R. Amiel, Jamie Havrilak, Bailey Steinworth, Taylor Chock, Eric Röttinger, Mark Q. Martindale

**Affiliations:** 1 Department of Biological Sciences, Lehigh University, Bethlehem, PA USA; 2 The Whitney Marine Laboratory for Marine Science, University of Florida, St. Augustine, Florida USA; 3 Université Nice Sophia Antipolis UMR 7284, CNRS UMR 7284, INSERM U1081, Institute for Research on Cancer and Aging, Nice, France

## Abstract

**Background:**

The nerve net of *Nematostella* is generated using a conserved cascade of neurogenic transcription factors. For example, *NvashA*, a homolog of the *achaete-scute* family of basic helix-loop-helix transcription factors, is necessary and sufficient to specify a subset of embryonic neurons. However, positive regulators required for the expression of neurogenic transcription factors remain poorly understood.

**Results:**

We show that treatment with the MEK/MAPK inhibitor U0126 severely reduces the expression of known neurogenic genes, *Nvath-like*, *NvsoxB(2)*, and *NvashA*, and known markers of differentiated neurons, suggesting that MAPK signaling is necessary for neural development. Interestingly, ectopic *NvashA* fails to rescue the expression of neural markers in U0126-treated animals*.* Double fluorescence in situ hybridization and transgenic analysis confirmed that *NvashA* targets represent both unique and overlapping populations of neurons. Finally, we used a genome-wide microarray to identify additional patterning genes downstream of MAPK that might contribute to neurogenesis. We identified 18 likely neural transcription factors, and surprisingly identified ~40 signaling genes and transcription factors that are expressed in either the aboral domain or animal pole that gives rise to the endomesoderm at late blastula stages.

**Conclusions:**

Together, our data suggest that MAPK is a key early regulator of neurogenesis, and that it is likely required at multiple steps. Initially, MAPK promotes neurogenesis by positively regulating expression of *NvsoxB(2)*, *Nvath-like*, and *NvashA*. However, we also found that MAPK is necessary for the activity of the neurogenic transcription factor *NvashA*. Our forward molecular approach provided insight about the mechanisms of embryonic neurogenesis. For instance, *NvashA* suppression of *Nvath-like* suggests that inhibition of progenitor identity is an active process in newly born neurons, and we show that downstream targets of *NvashA* reflect multiple neural subtypes rather than a uniform neural fate. Lastly, analysis of the MAPK targets in the early embryo suggests that MAPK signaling is critical not only to neurogenesis, but also endomesoderm formation and aboral patterning.

**Electronic supplementary material:**

The online version of this article (doi:10.1186/s12915-016-0282-1) contains supplementary material, which is available to authorized users.

## Background

Cnidarians (e.g., corals, sea anemones, and “jellyfish”) are the closest group of animals to the Bilateria (all bilaterally symmetrical animals such as vertebrates, flies, and nematodes), and are thus an important taxon to understand the origin and evolution of complex traits such as nervous systems [1]. *Nematostella vectensis* is a proven cnidarian model because it is easy to maintain in laboratory culture, its genome is sequenced and annotated, and multiple tools exist for functional genetic approaches [1–6].

The *Nematostella* nervous system comprises endodermal and ectodermal nerve nets [7, 8]. Neuronal cell bodies are arranged in a “salt-and-pepper pattern” such that individual neurons are scattered amongst non-neural cell types. Differentiating neurons are first detected in the late blastula stage before invagination of the presumptive endodermal plate [7, 9]. Salt-and-pepper expression of both *NvsoxB(2)* and *Nvath-like* (also called *Nvarp3*) are the earliest known neurally expressed genes and they define proliferative neural progenitor cells [9, 10]. Shortly after *Nvath-like* and *NvsoxB(2)* are detected, expression of post mitotic neural markers such as *NvashA* and *Nvelav1* is detected [7–9, 11]. *Nvelav1* is broadly expressed in a large number of neurons, though it is still unclear if it is a pan-neuronal marker in *Nematostella* [8]. Morpholino (MO)-mediated knockdown of either *NvsoxB(2)* or *Nvath-like* results in a loss of expression of both *Nvelav1* and the neural subtype marker *NvashA* [9, 10]*. NvashA* is expressed in a smaller number of developing neurons at embryonic stages [11]. Functional characterization of *NvashA* clearly demonstrated that it is necessary and sufficient to promote expression of the neural marker *Nvelav1* and a number of putative neural subtype markers [11]. Based on previous work, a reasonable model for *Nematostella* neurogenesis is that Notch activity selects *Nvath-like + NvsoxB(2) +* neural progenitors from a pool of naïve cells; daughters of those progenitor cells express additional neurogenic genes such as *NvashA*, which in turn promote expression of post mitotic markers such as *Nvelav1* and neural subtype markers [1]. However, the upstream inductive mechanisms responsible for initiating neurogenic cascades in *Nematostella* remain elusive, as do the molecular programs that give rise to *NvashA*-independent neural subtypes during neurogenesis.

FGF, Wnt, BMP, and Mitogen-activated protein kinase (MAPK) signaling cascades regulate neural induction in multiple species [12–17]. FGF, Wnt, and MAPK all promote neural development in other species [12–14, 16], whereas BMP activity is best known for its role in suppressing neural induction of the forming central nervous systems of model systems [14, 15, 18]. In *Nematostella* the role of these signaling pathways during neural induction is unclear. Disruption of Wnt signaling does result in neural phenotypes. However, the phenotypes are attributable to disrupted axial patterning [19–21]. Neural phenotypes resulting from loss and gain of BMP activity are complicated in that either manipulation results in loss of neurons, and neural phenotypes are restricted to larval stages [22, 23]. FGF-mediated MAPK signaling is one of many ways to initiate a receptor tyrosine kinase (RTK) cascade. Investigation of FGF signaling in *Nematostella* has primarily focused on its role in apical organ formation, and no broad neural phenotypes are reported, with the caveat that the current array of neural markers did not exist at the time of the initial study [24]*.* To date, the impact of MAPK signaling on neurogenesis has not been reported in *Nematostella.*

RTK signaling cascades are characterized by a series of kinases that are activated by upstream kinase and in turn activate a downstream kinase. Near the end of the cascade, activated MEK kinase phosphorylates ERK, which can translocate to the nucleus to phosphorylate and activate a number of transcription factors. Multiple RTK signaling cascades converge on MEK. For example, in *Nematostella*, FGF signaling controls apical organ formation at larval stages, and pharmacological inhibition of MEK is able to phenocopy gene-specific MO-mediated knockdown of the FGF-receptor *NvfgfRa* [24]. The number of MAPK-like pathways that could be acting to regulate neural development in *Nematostella* is large. There are at least 12 FGF-like ligands and two FGF receptors in the *Nematostella* genome [2, 25]. Two ligands and one receptor (*Nvfgfa1*, *Nvfgfa2*, *NvfgfRa*) are expressed in the vegetal hemisphere/apical domain, the ligand *Nvfgf8* is expressed in the animal hemisphere and its descendants, and the receptor *NvfgfRb* in derivatives of both poles [24–26]. There are at least 25 additional receptors (Johnston & Röttinger, unpublished) that could activate MEK/ERK signaling in *Nematostella*. Because the number of possible RTKs is relatively high, one strategy to better understand how these genes might be acting to regulate neurogenesis is to inhibit MEK. Thus, a number of possible signaling pathways can be simultaneously disrupted to determine if MAPK signaling contributes to neural development.

Here we use U0126, a potent and specific inhibitor of MEK [27], to test if MAPK signaling plays a role in neurogenesis, and to determine if we can use this disruption to identify other putative neural genes in the early embryo. We show that treatment with U0126 reduces expression of *Nvath-like*, *NvsoxB(2)*, *NvashA*, and post-mitotic neural markers. Loss of embryonic neurogenesis following U0126 treatment cannot be rescued by *NvashA*, suggesting U0126 treatment desensitizes embryonic cells from responding to proneural cues. We performed a genome-wide expression array to identify new MEK downstream targets, enabling us to characterize 18 novel salt-and-pepper-expressed genes. We tested the role of the putative FGF receptor *NvfgfRa*, which was the most likely candidate pathway disrupted by U0126 in regards to neural marker expression. However, we found no evidence suggesting that *NvfgfRa* signaling regulates neurogenesis or any of the salt-and-pepper genes identified in the U0126 microarray. We also investigated the relationship of these novel salt-and-pepper genes with *NvashA*-dependent neurogenesis*.* We confirmed one positive and one negative target of *NvashA*, *Nvvsx-like* and *Nvath-like* respectively*.* Lastly, we expanded our study to gain insight into whether *NvashA* regulated one or multiple neuronal subtypes. Using transgenic animals and double fluorescent mRNA *in situ* hybridization, we confirmed that *NvashA* regulates at least two distinct neural subtypes; however, based on its expression pattern at later developmental stages [11], this number is likely much higher. Additionally, the identification of ~80 genes that are expressed in the presumptive endomesoderm and aboral pole suggests that MAPK signaling plays a key role in multiple aspects of *Nematostella* embryogenesis. Taken together, our data and previously published results allow us to incorporate MEK, a key regulator, in a preliminary gene regulatory network describing embryonic *NvashA*-dependent neurogenesis, and provided us with a list of additional likely neurogenic genes, aboral genes, and endomesodermal genes for future studies.

## Results

### Determining ideal dose of U0126 to inhibit MEK inhibition

**Fig. 1:**
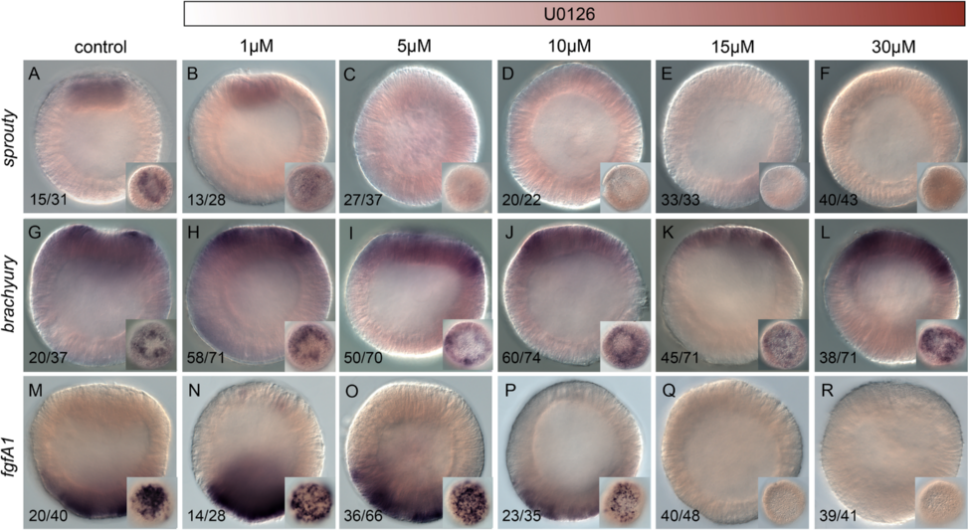
Dose-dependent effects of the MEK inhibitor U0126 on embryonic gene expression. Control blastula stages at 24 hours post fertilization (**a, g, m**) and embryos treated with increasing concentrations of U0126 (**b**–**f**, **h**–**l**, **n**–**r**). In situ hybridization on blastula stages using *Nvsprouty* (**a**–**f**), *Nvbrachyury* (**g**–**l**), or *NvfgfA1* (**m**–**r**) antisense probes. All images are lateral views with the presumptive endomesoderm (animal pole, future oral pole) to the top. The insets correspond to animal pole views. Numbers in lower left corner correspond to the number of animals with phenotype pictured / total number of animals assayed

**Fig. 2:**
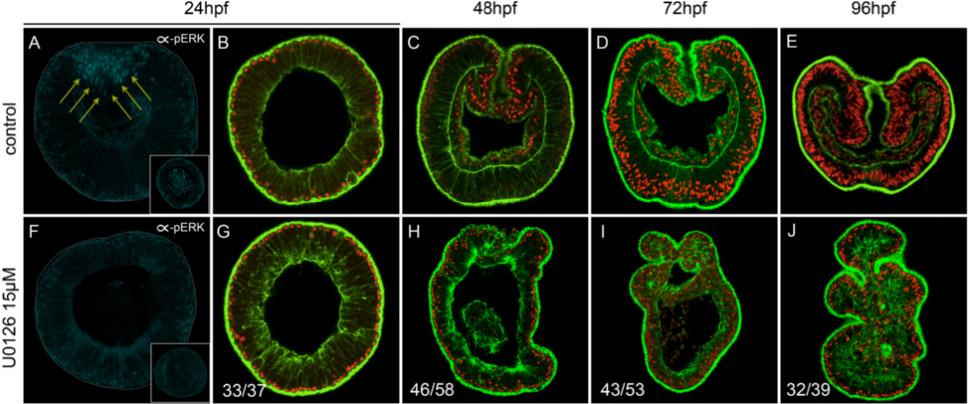
U0126 blocks ERK activation, endomesoderm formation, and gastrulation. **a**–**e** Control embryos. **f**–**j** U0126-treated embryos. **a**, **f** Confocal z-sections using anti-phospho-ERK (*cyan*) to visualize activated ERK. The *gray* dotted lines indicate the outline of the embryo and *yellow arrows* the accumulation of phosphorylated ERK (*pERK*)-positive cells above background levels. **b**–**e**, **g**–**j** Confocal z-sections using phalloidin (*green*) to show f-actin filaments and propidium iodide (*red*) to visualize the nuclei. **a**, **b**, **f**, **g** Blastula stages [24 hours post fertilization (*hpf*)]. **c**, **h** Late gastrula stages (48 hpf). **d**, **i** Early planula (72 hpf). **e**, **j** Late planula (96 hpf). All images are lateral views with the animal/oral pole to the top. The insets in **a** and **f** correspond to animal pole views. Ratios in **g**–**j** indicate the number of embryos displaying the phenotype shown in the image to the total number of analyzed embryos

To determine the most effective concentration of U0126 to use for our analyses, we treated fertilized zygotes with increasing concentrations of U0126 and analyzed expression of the previously described U0126 target *Nvfgfa1* as well as two markers of the animal hemisphere, *Nvsprouty* and *Nvbra* [24, 25, 28, 29] (Fig. 1). *Nvsprouty* was expressed throughout a central domain at the animal pole in the presumptive endoderm (Fig. 1a, whereas *Nvbra* was expressed in a central ring surrounding the future endodermal tissue (Fig. 1h). These two additional markers were chosen because we observed gastrulation failures in preliminary tests of the U0126 compound on early embryos and, in bilaterians, expression of *sprouty* homologs is downstream of FGF signaling (Fig. 2) [30]. U0126 treatments of 1–10 μM reduced *Nvsprouty* expression, but had little to no impact on the expression of *Nvfgfa1* or *Nvbra* (Fig. 1a–d, g–j, m–p; Additional file 1: Table S1). However, at a concentration of 15 μM, both *Nvfgfa1* and *Nvsprouty* were undetectable in U0126-treated animals (Fig. 1e, q; Additional file 1: Table S1). Interestingly, treatment with 15 μM U0126 induced ectopic *Nvbra* expression within the endoderm-forming central domain (Fig. 1k, l), suggesting that MEK signaling actively represses *Nvbra* in the most central endoderm-forming domain. Based on the ectopic expression of *Nvbra* in the central domain as well as the complete inhibition of *Nvsprouty* and *Nvfgfa1* expression in 15 μM treatments (Fig. 1e, k, q; Additional file 1: Table S1), we concluded that 15 μM of U0126 is an effective dose, and it is unlikely phenotypes are due to toxicity.

### Inhibition of MEK prevents ERK phosphorylation and gastrulation

To ensure U0126 treatment is inhibiting MEK, we screened embryos treated with either dimethyl sulfoxide (DMSO) as a control or U0126 for the presence of phosphorylated ERK (pERK) (Fig. 2) [31]. pERK staining in controls was detected at low levels in most cells of the blastula, but was enriched in the presumptive endoderm cells that undergo invagination (Fig. 2a). In U0126 treatments, a positive pERK signal above the general ectodermal staining was not detected (Fig. 2f). We further analyzed the morphological phenotypes induced by the disruption of ERK signaling and observed that treated embryos dramatically failed to gastrulate and form a gut (Fig. 2g–j) compared to control embryos (Fig. 2b–e).

### *NvashA*, *Nvath-like*, and *NvsoxB(2)* are globally downregulated in U0126-treated embryos

**Fig. 3:**
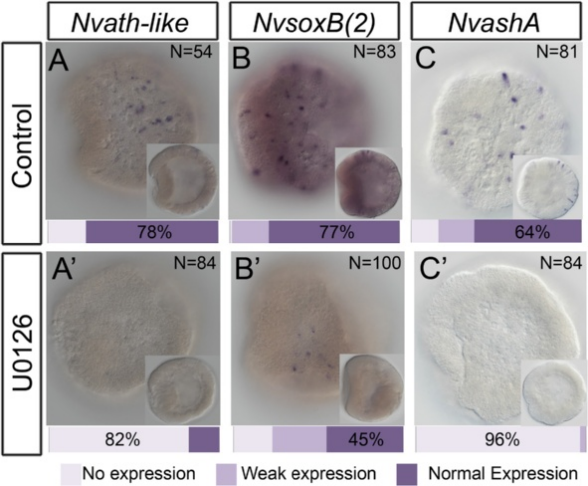
U0126 treatment results in a global decrease in neurogenic transcription factors. mRNA *in situ* expression of *Nvath-like* (**A**), *NvsoxB(2)* (**B**), and *NvashA* (**C**) in control embryos treated with in 1/3× artificial seawater (ASW) with 0.1 % dimethyl sulfoxide (DMSO). Expression of *Nvath-like* (**A’**), *NvsoxB(2)* (**B’**), and *NvashA* (**C’**) in animals treated with 15 μm U0126 in 1/3× ASW with 0.1 % DMSO. Embryos were classified and quantified as the percent having normal expression, weak expression, or no expression. Refer to key in figure for classification of phenotypes. The phenotypic class with the highest percentage of embryos is indicated. All embryo images are of early gastrula stage. The main figure panels are ectodermal focal planes of lateral views with the presumptive oral side to the left. The insets show deeper focal planes used to confirm embryonic stage

We next tested if U0126 treatment would disrupt expression of the known neural genes. We treated embryos with U0126 and scored for expression of known neural genes *NvashA*, *Nvath-like*, and *NvsoxB(2)* (Fig. 3). mRNA *in situ* hybridization on U0126-treated and control animals during early gastrula stages [24 hours post fertilization (hpf) at 17 °C] (Fig. 3) revealed that all three genes—*NvashA*, *NvsoxB(2)*, and *Nvath-like*—were globally reduced in U0126-treated animals (Fig. 3). However, they did not all display the same sensitivity to U0126. *Nvath-*like expression was undetectable in 82 % and *NvashA* expression was undetectable in 96 % of U0126-treated embryos (Fig. 3A, C). *NvsoxB2* expression was dramatically reduced, both in terms of the number of cells expressing it and in the level of expression (Fig. 3B), but expression was detectable in many more embryos than observed for either *NvashA* or *Nvath-like*. Interestingly, we identified *NvsoxB(2)* as being maternally expressed using the SeaBase database of transcriptomes (Additional file 2: Figure S1) [32–34]. To address whether or not U0126 might have a more severe impact on *NvsoxB(2)*, we treated animals at late gastrula stages for 24 hours with U0126 or DMSO control. We observed that *NvsoxB(2)* expression was not detectable in 87 % of U0126-treated cells (Additional file 3: Figure S2A). We also observed a strong reduction in *NvashA* expression in animals treated with U0126 for 24 hours after completion of gastrulation (Additional file 3: Figure S2B). These data suggest that the earliest known neurogenic transcription factors are globally reduced in U0126-treated animals, which indicates that neurogenesis is disrupted by U0126.

### *NvashA* misexpression fails to rescue neurogenesis in U0126-treated animals

**Fig. 4:**
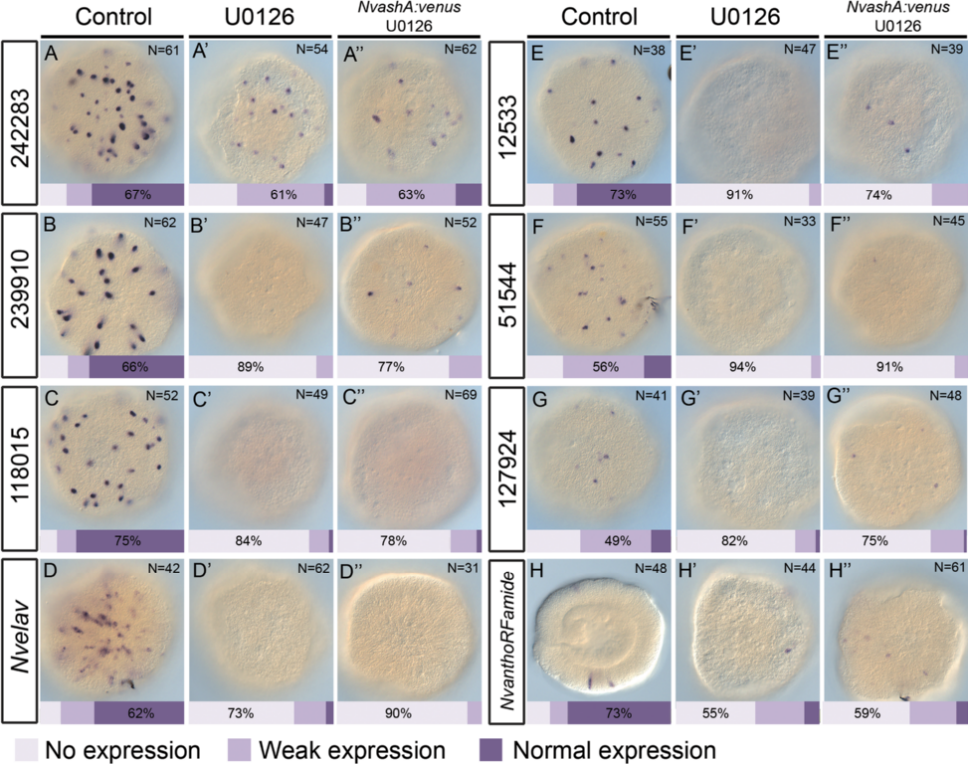
*NvashA* is insufficient to rescue neuronal loss resulting from U0126 treatment. mRNA *in situ* expression of each gene is indicated in control embryos raised in 1/3× artificial seawater (ASW) with 0.1 % dimethyl sulfoxide (DMSO) (**A**–**H**), compared to embryos treated with 15 μm U0126 in 1/3× ASW with 0.1 % DMSO (**A’**–**H’**), and compared to embryos injected with *NvashA:venus* mRNA and treated with 15 μm U0126 in 1/3× ASW with 0.1 % DMSO (**A”**–**H”**). Genes are referred to by either name or protein ID number used in the *Nematostella genome* database v1.0 (http://genome.jgi.doe.gov/Nemve1/Nemve1.home.html). Embryos were classified and quantified as the percent having normal expression, weak expression, or no expression. The phenotypic class with the highest percentage of embryos is indicated. Treatment with U0126 strongly reduced all neural gene expression (compare **A’**–**H’** with **A**–**H**). Misexpression of *NvashA* in U0126-treated animals was not sufficient to rescue neuronal loss induced by U0126 treatment (compare **A”**–**H”** to both **A**–**H** and **A’**–**H’**). All animals in panels **A**–**G**, **A’**–**G’**, and **A**–-**G”** are aboral ectodermal focal planes of 48 hours post fertilization embryos (late gastrula stage). Embryos in panels **H**, **H’**, and **H”** are a lateral view with oral side facing towards the left

To test if U0126 treatment impacted neurogenesis, treated animals were allowed to develop until a time equivalent to the late gastrula stage and screened for expression of the broadly expressed neural marker *Nvelav1* and previously identified *NvashA* neural target genes by mRNA *in situ* hybridization. U0126 treatment dramatically reduced expression of neural markers, such that they were essentially undetectable (Fig. 4). Two exceptions were *NvLWamide-like* (PrtID# 242283, http://genome.jgi.doe.gov/Nemve1/Nemve1.home.html) and *NvanthoRFamide*, which showed staining in a few cells (Fig. 4A’, 4H’), but both were reduced compared to control DMSO-treated animals. We conclude that treatment with U0126 results in a nearly complete loss of embryonic neural marker expression.

We next aimed to determine if loss of neural markers in U0126-treated animals could be rescued by misexpression of neurogenic transcription factors. *NvsoxB(2)* and *Nvath-like* act upstream of *NvashA*, both have much broader roles than *NvashA* in neurogenesis, and both are expressed in neural progenitor cells [9, 10]. However, misexpression phenotypes are not reported for either of these genes. We were also concerned that over-activation of progenitor cell markers could result in a loss of neural markers due to an inability to transition from an undifferentiated to a differentiated state [35]. Thus, observing no rescue with misexpression of either *NvsoxB(2)* or *Nvath-like* could represent a false negative neural phenotype. *NvashA* is expressed in differentiated cells, and misexpression of *NvashA* has already been shown to be sufficient to induce ectopic neural marker expression [9, 11]*.* We injected in vitro transcribed *NvashA:venus* mRNA and treated animals with U0126. Surprisingly, we observed no rescue of the neural markers (Fig. 4A"–H”). To ensure that *NvashA:venus* behaves as previously reported [6, 11], we treated animals injected with *NvashA:venus* mRNA with U0126. High levels of NvAshA:Venus were still detectable and localized to the nucleus (Additional file 4: Figure S3). We conclude that *NvashA* is not sufficient to rescue neural marker expression in U0126-treated animals.

The failure of *NvashA* to rescue U0126-induced loss of neurogenesis might be a consequence of the reduced *NvsoxB(2)* and *Nvath-like* expression in U0126-treated animals. To address this hypotheses we took advantage of the previous observation that *NvashA* restores neural marker expression lost in animals with hyperactivated Notch signaling even though *NvsoxB(2)* and *Nvath-like* remain strongly downregulated [36]. To compare the downregulation of *NvsoxB(2)* and *Nvath-like* induced by hyperactivation of Notch to the downregulation observed in U0126-treated animals, we performed mRNA in situ hybridization experiments in *NvnotchICD:venus*-injected animals (Additional file 3: Figure S2). Injection of *NvnotchICD:venus* mRNA resulted in 83 % of animals showing no or weak *NvsoxB(2)* expression and 88 % of animals showing no *Nvath-like* expression (Additional file 3: Figure S2). Both genes were more severely reduced in *NvnotchICD:venus*-injected animals than they had been in U0126-treated animals (compare Fig. 3A, B to Additional file 3: Figure S2A, B). Taken together with previous reports that *NvashA* rescues neuronal loss induced by Notch hyperactivity without increasing *NvsoxB(2)* or *Nvath-like* expression, we argue that U0126 disrupts neurogenesis in two ways. It inhibits the expression of the neurogenic transcription factors *Nvath-like*, *NvsoxB(2)*, and *NvashA*, and it disrupts a yet unknown pathway that is also required for *NvashA* to promote neural fates.

### *NvashA* target genes likely represent multiple distinct neuronal subtypes

**Fig. 5:**
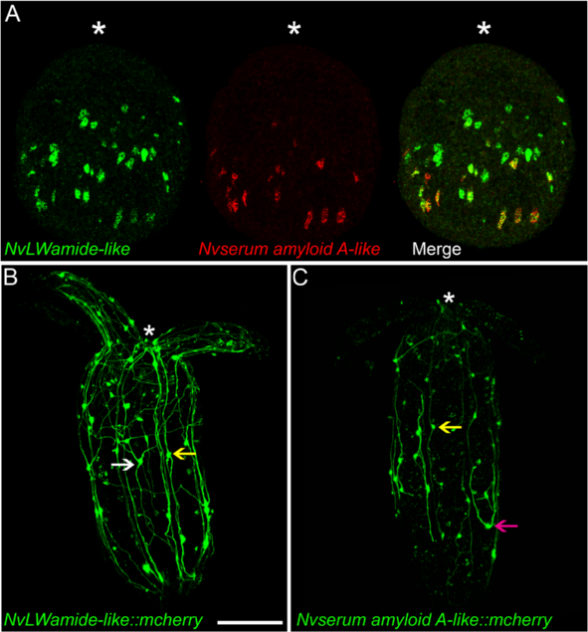
*NvashA* regulates multiple neuronal subtypes. Shown are three-dimensional projections of two juvenile polyps shown with the oral side towards the top of the image. **a** Double fluorescent in situ hybridization of *NvLWamide-like* (*red*) and *Nvserum amyloid A-like* (*green*) in a late gastrula stage embryo. **b** The transgenic line for *NvLWamide::mcherry* expression is shown. Neural soma and neurites are observed throughout the body column and tentacles. Ectodermal neurons with three projections are observed in the body column (*white arrow*), which are not found in the other transgenic line. **c** The transgenic line for *Nvserum amyloid A-like::mcherry* is shown. There are many fewer neurons compared to *NvLWamide::mcherry*, but characteristic neurons are present. The U-shaped neuron that has two orally projecting neurites (*pink arrow*) is specific to this transgenic line. Both lines have neurons that are located just over the mesenteries and send projections orally and aborally in neural tracts overlaying the mesenteries (*yellow arrows*). In all images, asterisks indicate relative position of mouth

We wanted to confirm that *NvashA* regulates multiple neuronal subtypes. Although *NvashA* is expressed in a subset of the nervous system, subtle differences in the expression domains of *NvashA* targets suggests that they describe distinct neural subtypes [11]. To test if *NvashA* regulates distinct neural subtypes we performed double fluorescent in situ hybridizations and created stable meganuclease-mediated transgenic reporters for two of the *NvashA* targets using an approximately 2000-base-pair genomic region immediately upstream of the start codon for both genes to drive expression of mCherry fluorescent protein (Fig. 5) [37]. We chose *NvLWamide-like* (PrtID# 242283) (Fig. 5a, b) and *Nvserum amyloid A-like* (PrtID# 239910) (Fig. 5a, c) because these genes represent *NvashA* targets with overlapping but slightly different expression patterns during development, and both genes were strongly downregulated in the U0126 microarray (see below; Additional file 5: Table S2, Additional file 6: Table S3). Both genes were expressed broadly throughout the aboral region of the embryo, but the *NvLWamide-like* expression domain extended more orally and encompassed a larger domain than that of *Nvserum amyloid A-like* (Fig. 5a) [11]*. NvLWamide-like* was expressed in more cells than *Nvserum amyloid A-like* (Fig. 5a). Double fluorescent mRNA in situ hybridizations revealed that many of the *Nvserum amyloid A-like* expressing neurons (Fig. 5a, red) were also positive for *NvLWamide-like* (Fig. 5a, green). However, there were many examples of *NvLWamide-like*-only-positive cells and few examples of *Nvserum amyloid A-like*-only-positive cells (Fig. 5a). Thus, it is likely that there are at least three molecularly distinct neural subtypes that require *NvashA* for development.

We next compared the neurons labeled in each transgene to determine if the neurite projections from *Nvserum amyloid A-like* and *NvLWamide-like* neurons are similar or distinct. Although there were a number of neurons with similar neurites that projected along the oral–aboral axis in each transgene (Fig. 5b, c, yellow arrows), each transgene also labeled neurons with distinct morphologies (Fig. 5b, c, white and pink arrows, respectively). Consistent with the mRNA in situ hybridization results, the NvLWamide-like::mCherry labeled more cells than NvSerum Amyloid A-like::mCherry (compare Fig. 5b and c). Interestingly, there were distinct cell types present in each line [for example, NvLWamide-like::mCherry was expressed in ectodermal cells that had three neurites extending from the soma (Fig. 5b, white arrow), and NvSerum Amyloid A-like had unique neurons with neurites that extended out of either side of the soma and projected orally to form U-shaped neurons (Fig. 5c, pink arrow)] that could be reproducibly identified in individual transgenic animals. Although more extensive characterization is required for each of these transgenic lines, the fact that *NvashA* targets have distinct expression patterns and that neurons described by the transgenes of two *NvashA* targets display distinct morphologies support the conclusion that *NvashA* regulates multiple neuronal subtypes.

### Identification of 100 genes downregulated and 22 genes upregulated by U0126 treatments

To determine if U0126 regulates other potential neurogenic genes and to identify novel targets of MEK signaling, we applied a forward molecular approach using a genome-wide expression microarray. Zygotes were treated with U0126 or DMSO until late blastula stage. At that point mRNA was extracted and used to generate labeled cDNA, which was hybridized to a custom expression microarray (Nimblegen, Inc.) that represents 24,021 predicted *N. vectensis* gene models [28]. The Pearson’s correlation factor between biological replicates was mediocre (0.69), however, 926 genes were significantly (*P* < 0.05) upregulated and 1176 genes were significantly (*P* < 0.05) downregulated in U0126-treated embryos (Additional file 6: Table S3). Because our focus was to identify early embryonic patterning events, we screened the lists of genes to identify transcription factors, signaling molecules, and signaling modulators. This resulted in the identification of 100 genes that were downregulated and 22 genes that were upregulated by U0126 (Additional file 7: Table S4; Additional file 8: Table S5 [2, 7, 9, 11, 19, 24, 26, 28, 38–57]). Out of the 122 selected genes, the vast majority (86/122) possess strong orthology with members of various families of transcription factors (i.e., forkhead, pointed, homeodomain). Twenty-seven genes potentially encode ligands, modulators, or receptors of signaling pathways (i.e., Wnts), and nine genes show similarities with adhesion molecules, metalloproteases, or RNA binding proteins with described developmental functions in bilaterians (i.e., Ncam, Tolloid, Vasa) (Additional file 6: Table S3). To distinguish between previously published genes and newly identified ones, we used the best Blast Hit identification followed by “*-like*”. Thus, with the exception of *Nvhes-like* and *Nvath-like* [9, 51], all gene names containing “*-like*” designate gene products described for the first time in this study.

### Identification of putative neural genes

**Fig. 6:**
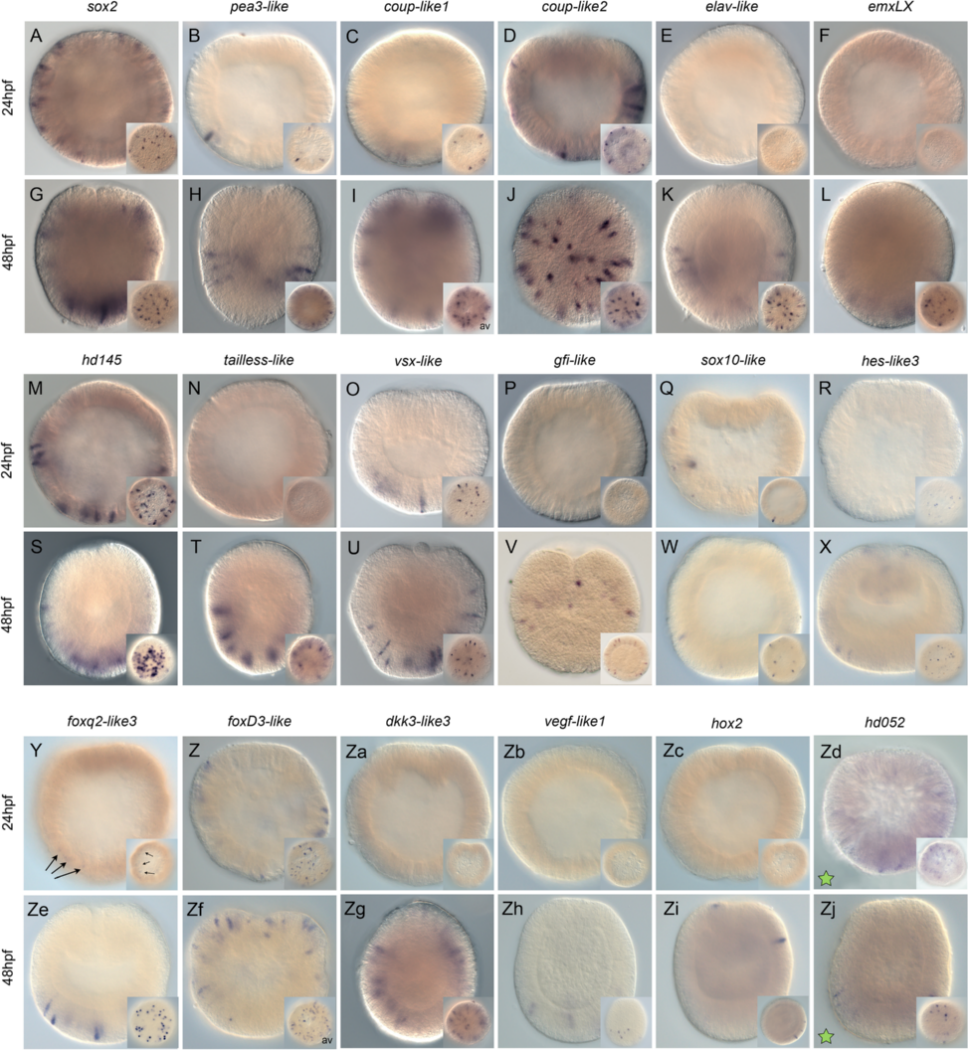
U0126 targets expressed in individual cells throughout the ectoderm. Wild type salt-and-pepper gene expression analysis by in situ hybridization of genes differentially regulated by U0126 treatments. All animals are either blastula [24 hours post fertilization (*hpf*) – **A**–**F**, **M**–**R**, **Y**-**Zd**] or gastrula (48 hpf – **G**–**L**, **S**–**X**, **Ze**–**Zj**) stages. All images are lateral views with the animal pole to the top. The insets correspond to surface views. Antisense probes used as indicated. *Green stars* in Zd and Zj indicate that this gene was upregulated under U0126 conditions. All other genes were downregulated

Neural genes are predicted to have a salt-and-pepper expression pattern, and thus determining the expression pattern of the 122 target genes would identify which genes exhibit salt-and-pepper expression associated with neural genes. We performed whole mount in situ hybridizations in the developing embryo at blastula stages (24 hpf) and at the end of gastrulation (48 hpf, Fig. 6). We excluded 16 genes because their expression patterns are already described at the blastula stage, but we did include genes whose early expression patterns had not yet been described (*Nvgata*, for example [23]). We were able to obtain clones for 98 of the remaining 106 genes and synthesized anti-sense probes. All original publications corresponding to a given gene (sequence identification and/or gene expression pattern) can be found in Additional file 7: Table S4 and Additional file 8: Table S5. Our in situ screen revealed reproducible patterns for 60 of the 98 genes we screened. Patterns could be grouped into one of three categories: (1) genes expressed in the animal hemisphere/presumptive endomesoderm of the blastula (24/98) (Additional file 9: Figure S4); (2) genes expressed in the vegetal hemisphere/aboral domain of the blastula (18/98) (Additional file 9: Figure S4); and (3) genes with the characteristic salt-and-pepper pattern consistent with being neural genes (18/98) (Fig. 6). We found no link between genes that are expressed broadly in the animal and vegetal hemispheres and neurogenesis, and thus will not discuss these genes further here.

### Genes expressed in individual cells throughout the ectoderm

The 18 genes expressed in individual cells of the presumptive ectoderm at the blastula stage or in ectodermal body wall at the end of gastrulation were *Nvsox2*, *Nvpea-like3*, *Nvcoup-like1* (also called NR-like 12), *Nv-coup-like2* (also called NR-like 13) [55], *Nvelav-like*, *NvemxLX*, *Nvhd145*, *Nvtailless-like*, *Nvvsx-like*, *Nvgfi-like*, *Nvsox10-like*, *Nvhes-like3*, *Nvfoxq2-like3*, *NvfoxD3-like*, *Nvdkk-like3*, *Nvvegef-like1*, *Nvhox2*, and *Nvhd052* (Fig. 6). Eight genes (*Nvelav-like*, *NvemxLX*, *Nvtailless-like*, *Nvgfi-like*, *Nvdkk3-like3*, *Nvvegf-like1*, and *Nvhox2 and Nvhd052 we did not detect localized expression prior to the onset of gastrulation* (Fig. 6E,F,N,P,Za-Zd). Six genes (*Nvpea3-like*, *Nvcoup-like1*, *Nvvsx-like*, *Nvsox10-like*, *Nvhes-like3*, and *Nvfoxq2-like3*) were expressed in 5–15 cells (Fig. 6B, C, O, Q, R, Y). For four genes (*Nvsox2*, *Nvcoup-like2*, *Nvhd145*, and *NvfoxD3-like*), more than 15 cells were stained throughout the presumptive ectoderm at the blastula stage (Fig. 6A, D, M, Z).

At the end of gastrulation, we observed gene expression for all above-mentioned genes in individual cells (Fig. 6G–L, S–X, Ze–Zj, Zl). However, the localization and number of stained cells varied considerably. The only gene that appeared to be expressed in individual cells throughout the entire ectoderm (pharyngeal ectoderm, oral ectoderm, body wall ectoderm, sub-apical pole, and apical pole) was *Nvhes-like3* (Fig. 6X), which is reminiscent of a previously reported gene, *NvashA* [11, 51]. *Nvsox2* and *Nvcoup-like1* (Fig. 6G, I) were also expressed in a salt-and-pepper manner throughout all ectodermal domains, except the pharyngeal ectoderm. The largest group of genes (*Nvcoup-like2*, *Nvelav-like*, *NvemxLX*, *Nvhd145*, *Nvtailless-like*, and *Nvvsx-like*) was detected in the body wall ectodermal as well as the sub-apical and apical domains (Fig. 6J, K, L, S, T, U). Cells expressing *NvfoxD3-like* and *Nvdkk3-like3* (Fig. 6Zf, Zg) were detected in the oral and body wall ectoderm as well as the sub-apical pole domains; however, *Nvsox10-like*, *Nvfoxq2-like3*, and *Nvvegf-like* were only detected in the body wall ectoderm and sup-apical domain (Fig. 6W, Ze, Zh). The genes with the most restricted expression domain were *Nvpea3-like*, *Nvgfi-like*, and *Nvhox2*, which were expressed in individual cells either within a circumferential territory (Fig. 6H, V) or in a patch within the body wall ectoderm (Fig. 6Zi), respectively. The variable expression patterns suggest that many of these genes are putatively expressed in distinct subsets of neurons, while a few broadly expressed genes might play larger roles during neural development.

### Temporal gene expression analysis of salt-and-pepper genes

In situ hybridization provides crucial spatial information about gene expression but cannot be used quantitatively to determine the presence of maternal transcripts or zygotic upregulation of a given gene. However, this information is crucial for the design of functional studies, to predict potential genetic interactions, and to build gene regulatory networks [28]. In order to determine the temporal deployment of putative neural genes during early embryogenesis, we performed fine-scale quantitative reverse transcription PCR (RT-qPCR) on RNA/cDNA that was sampled from unfertilized eggs and every 2–4 h during embryogenesis up to the late gastrula stage (48 hpf) Additional file 10: Figure S5. We analyzed 21 salt-and-pepper genes identified both in this and in previous studies, including the known neural genes such as *Nvath-like* and *NvashA* [9, 11]. Five salt-and-pepper genes (*Nvfoxq2-like3*, *Nvcoup-like2*, *Nvelav-like*, *NvpaxA*, and *Nvvegf-like1*) were detectable in zygotes, indicating that they are maternal genes (Additional file 11: Figure S6). However, it is not yet clear what the significance of these genes is because none of these genes have broad expression patterns that might indicate a key early role for them in neurogenesis. The majority of salt-and-pepper genes, including the known neural regulators *Nvath-like* and *NvashA*, are not components of the maternally contributed mRNAs, which suggests that the earliest neural fates are induced in the embryo. As a group, the salt-and-pepper genes display a similar temporal deployment. The earliest zygotically regulated genes are *Nvath-like* (8–10 hpf), followed by *Nvhes3* (10–12 hpf), *NvfoxD3*, *Nvsox10-like* (12–14 hpf), *Nvfoxq2-like3*, and *Nvsox2* (16–18 hpf), and then the bulk of genes (12/21) are upregulated at either 18–20 hpf (*Nvcoup-like1*, *Nvcoup-like2*, *Nvgfi-like*, *Nvhd145*, *Nvtailless-like*, *Nvvsx-like*, and *Nvelav-like*) or 20–24 hpf (*Nvdkk-like3*, *Nvpea3-like*, *NvpaxA*, *Nvgcm*, and *NvashA*). Our temporal analysis demonstrated two key aspects. *Nvath-like*, which is the first neural gene to respond to treatment with DAPT and is thought to be the earliest acting neural gene [9], is in fact upregulated prior to other known neural genes. Second, the salt-and-pepper genes all showed strong upregulation between 10 and 24 hpf. The upregulation of genes is continuous in that different genes are upregulated at different times throughout the 10–24 hpf window. This argues that once salt-and-pepper gene expression is initiated there is a steady increase in the expression of distinct salt-and-pepper genes. Additionally, temporal differences in expression might reflect a hierarchal organization for their functions. The later concept is supported by the observation that *Nvath-like* (an upstream neural regulator) is detected much earlier than *NvashA*, which is expressed in differentiated neurons [9].

#### *NvFGFRa* does not regulate salt-and-pepper gene expression in the embryo

**Fig. 7:**
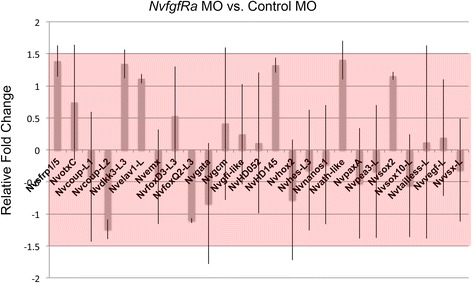
*NvfgfRa* does not regulate U0126-dependent salt-and-pepper gene expression. Relative fold change calculated from quantitative polymerase chain reaction analysis of triplicate injections of the *NvfgfRa* morpholino (*MO*) or a control MO is graphed. Broadly expressed genes, *Nvsfrp1/5*, *NvotxC*, and *NvfgfRa*, are included as controls*.* The remaining genes are the salt-and-pepper expressed genes identified by the U0126 microarray. The *red box* indicates a region (1.5× to −1.5× fold change) that was defined as the cut off for a significant change in expression. Error bars represent standard error

ERK-mediated FGF signaling can be inhibited by treatment with U0126 in *Nematostella* [24], and the *NvfgfRa* receptor is expressed broadly in the embryonic ectoderm, which makes it a likely candidate responsible for the U0126 neural phenotype and/or responsible for regulating some of the newly identified salt-and-pepper genes. We tested if *NvfgfRa* might have an early neural phenotype by injecting the previously described *NvfgfRa* MO into embryos [24] and scoring for changes in *Nvath-like*, *NvashA*, and the other 19 genes with salt-and-pepper gene expression identified in the U0126 microarray. qPCR analysis comparing control MO and *NvfgrRa* MO revealed that none of the salt-and-pepper genes responded to changes in *NvfgfRa* levels (Fig. 7). These data suggest NvFgfra signaling is not the U0126 target responsible for the loss of salt-and-pepper expressed genes in the early gastrula. However, there is a maternal contribution of *Nvfgfra* signaling genes [24]. To confirm that this FGF signaling was unlikely the source of the neural phenotype, we allowed morphant animals to grow to 48 hpf at 17 °C. In parallel, we allowed wild-type animals to grow until 24 hpf, when they are approximately at the late blastula/early gastrula stage. Animals were then treated with U0126 from 24 to 48 hpf. This treatment regimen allows for the maternal action of FGF components, thus mimicking a potential flaw in the *Nvfgfra* morphant approach. qPCR analysis of *NvashA* and *Nvfgfra* (a target of *Nvfgfra* MO [24]) demonstrated that *Nvfgfra* MO effectively inhibited known targets, but not *NvashA* (Additional file 12: Figure S7B). We saw similar results with SU5402 treatment (Additional file 12: Figure S7B). However, U0126 treatment resulted in a strong reduction in all genes assayed (Additional file 12: Figure S7). These data argue that FGF signaling is not the source of the neural phenotype. Thus, although the neural phenotype observed in U0126-treated animals is robust, it is still unclear what signaling pathway(s) targeted by U0126 regulates salt-and-pepper gene expression in the embryonic ectoderm.

#### *NvashA* regulates a small subset of the salt-and-pepper genes

**Fig. 8:**
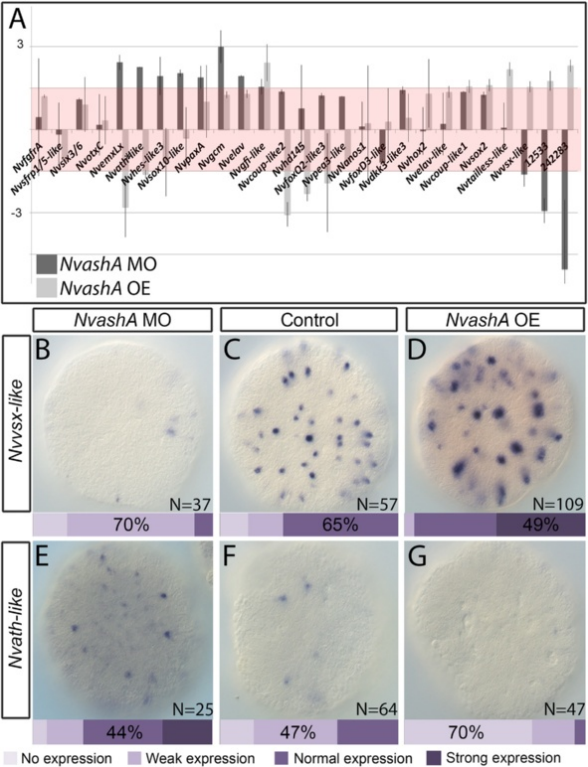
*NvashA* regulates a subset of U0126-dependent salt-and-pepper expressed genes. **a** Relative fold change calculated from quantitative polymerase chain reaction analysis of triplicate injections of the *NvashA* morpholino (*MO*) versus a control MO (*dark gray bars*), or *NvashA:venus*-injected versus control *venus*-injected animals (*light gray bars*). Broadly expressed *NvfgfRa*, *Nvsfrp1/5*, *Nvsix3/6*, and *NvotxC* regional patterning genes were included as controls*.* Two positive control genes, 12533 and 242283, are included. The remaining genes are the salt-and-pepper expressed genes identified by the U0126 microarray. The *red box* indicates a region between 1.5× and −1.5× fold change that corresponds to an insignificant change in expression. The reciprocal phenotypes observed in *NvashA* MO and *NvashA:venus* mRNA-injected animals for *Nvvsx-like* (**b**–**d**) and *Nvath-like* (**e**–**g**) were confirmed by mRNA in situ hybridization. Embryos were classified and quantified as the percent having normal expression, weak expression, or no expression. The phenotypic class with the highest percentage of embryos is indicated. All embryo images are at the early gastrula stage. All images are ectodermal focal planes of aboral views. Error bars represent standard error

We next wondered if *NvashA* acted to regulate any of the U0126 target genes. Based on the role of proneural genes in other animals we hypothesized that *NvashA* might act upstream of or suppress expression of subsets of the salt-and-pepper expressed U0126 targets. *NvashA* was knocked down or overexpressed using the previously described *NvashA* translation blocking MO and in vitro synthesized *NvashA:venus* mRNA [11]. We assayed U0126 salt-and-pepper target expression by qPCR after disruption of *NvashA* and confirmed any genes that showed a response to *NvashA* disruption by mRNA in situ hybridization (Fig. 8). We included six control genes. Four negative controls (*Nvfgfra*, *Nvsfrp1/5-like*, *Nvsix3/6*, and *NvotxC*) represented broadly expressed regional patterning genes unlikely to respond to changes in *NvashA* function [19, 24, 54]. Two positive control genes, *NvLWamide-like* (PrtID# *242283*) and canalicular multispecific anion transporter (PrtID# 12533), are both confirmed positive targets of *NvashA* [11] and were both also downregulated in the U0126 array (Additional file 5: Table S2).

We first assayed for positive targets of *NvashA.* Overexpression of *NvashA* increased the expression of *Nvvsx-like*, *Nvgfi-like*, *Nvcoup-like1*, *Nvsox2*, and *Nvtailless-like* (Fig. 8a, light gray bars). However, *Nvvsx-like* expression was weakly upregulated following injection of *NvashA:venus* mRNA (Fig. 8a light grey bars; Fig. 8d), but *Nvvsx-like* was the only upregulated gene that decreased after *NvashA* knockdown by MO injection (Fig. 8a, dark gray bars; Fig. 8b). These data argue that *NvashA* is necessary and sufficient for the expression of *Nvvsx-like* (Fig. 8a–d)*. NvashA* was sufficient but not necessary to regulate *Nvtailless*-*like* in the early gastrula. Overexpression of *NvashA* increased the expression of *Nvtailless-like* (Fig. 8a, light gray bars)*.* However, *Nvtailless* expression was not dependent on *NvashA*, as its expression level was not decreased in *NvashA* morphants (Fig. 8a, dark gray bars). *Nvgfi-like*, *Nvcoup-like1*, and *Nvsox2* also all showed increases in expression following injection of *NvashA* mRNA (Fig. 8a, light gray bars). However, injection of *NvashA* MO also resulted in increased expression of all three genes (Fig. 8a, dark gray bars). Because reciprocal phenotypes are not observed between mRNA-injected and MO-injected animals, and both morphant and overexpression phenotypes were similar, it is unclear if changes in expression of *Nvcoup-like1*, *Nvsox2*, and *Nvgfi-like* reflect normal *NvashA* activity. Thus, we exclude these genes as targets of *NvashA* until *NvashA* mutant analysis can confirm that they are downstream of *NvashA* in the early gastrula*.* We conclude that *Nvvsx-like* is a positive target of *NvashA*.

We next investigated the 10 genes, *Nvath-like*, *NvemxLx*, *Nvhes-like3*, *Nvsox10-like*, *NvpaxA*, *Nvgcm*, *Nvgfi-like*, *Nvhd145*, *Nvcoup-like2*, and *Nvelav1*, that displayed changes in expression consistent with being putative negative targets of *NvashA. Nvhd145* and *Nvcoup-like2* expression was reduced in *NvashA*-overexpressing animals, but no change in either gene was observed in *NvashA* morphants (Fig. 8a; Additional file 4: Figure S3). These data suggest that although *NvashA* is sufficient to suppress *Nvhd145* and *Nvcoup-like2*, it does not likely regulate these genes during embryonic stages. *Nvath-like*, *NvemxLx*, *Nvhes-like3*, *Nvsox10-like*, *NvpaxA*, *Nvgcm*, *Nvgfi-like*, and *Nvelav1* showed increased expression at early gastrula stages in *NvashA* morphant animals (Fig. 8a, light gray bars), which suggests that they might be negative targets of *NvashA*. Only *Nvath-like* and *NvemxLx* showed reciprocal changes in expression in *NvashA* gain and loss of function (Fig. 8a, dark gray bars versus light gray bars). We were only able to confirm the changes in *Nvath-like* expression by mRNA in situ hybridization (Fig. 8e–g). In situ hybridizations with the *NvemxLx* probe on wild-type embryos often took >2 weeks to develop, and even then it was only detectable in very few cells and in only a few of the animals, arguing that it is expressed at low levels and that mRNA in situ hybridization is not sensitive enough to verify this gene. Relative expression strength can be inferred by the qT value obtained for any given gene during qPCR analysis. Essentially, qT values above 35 are often associated with genes that are not expressed. We consistently obtained qT values of 32–33 for *NvemxLx*, arguing that it is in fact very weakly expressed. Although we could not confirm *NvemxLx* by mRNA in situ hybridization, it is likely a negative target of *NvashA* because it reproducibly showed reciprocal changes in expression in response to an increase or decrease in *NvashA* function (Fig. 8a). The remaining putative negative targets did not show reduced expression levels in *NvashA:venus* mRNA-injected animals, and with exception to *Nvsox10-like*, their increased expression in *NvashA* morphant animals could not be confirmed by mRNA in situ hybridization (Additional file 13: Figure S8; data not shown). The increased expression of *Nvelav1* in *NvashA* morphants was somewhat surprising, because it has already been shown to be a positive target of *NvashA* when assayed at later stages [11, 36]. However, *NvashA* only regulates a subset of *Nvelav1*-positive neurons [9–11], and thus we suspect that *NvashA* does not play a significant, if any, role in *Nvelav1* regulation at this early time point. We conclude that *Nvath-like*, *NvemxLX*, and *Nvsox10-like* are normally suppressed by *NvashA* at embryonic stages, and that *NvashA* is sufficient to suppress expression of *Nvath-like* and *NvemxLX* but not *Nvsox10-like*.

## Discussion

### Preliminary gene regulatory network and model describing *NvashA*-dependent neurogenesis in the embryonic ectoderm

**Fig. 9:**
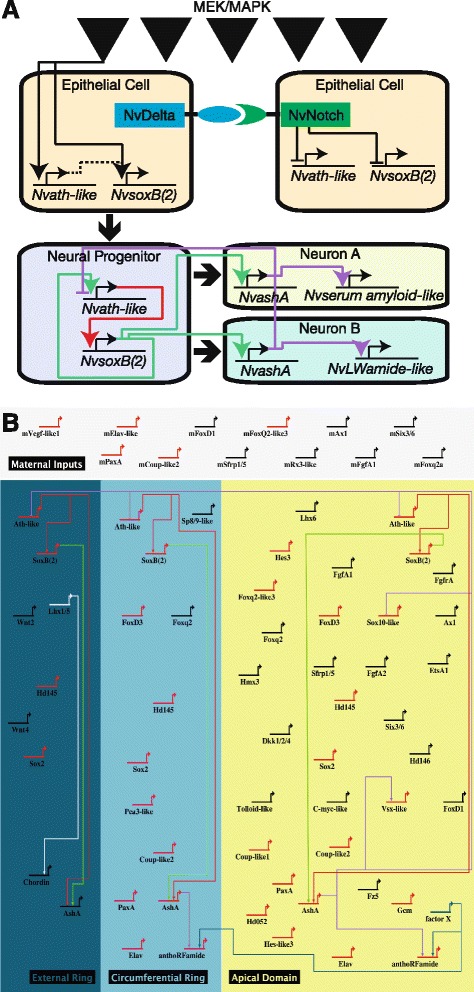
Model of *NvashA*-dependent embryonic neurogenesis. **a** Model describing *NvashA*-dependent neurogenesis at early gastrula stage. *Boxes* represent indicted cell types. *Solid regulatory lines* represent published observations, and *dashed lines* represent likely regulatory interactions. **b** Biotapestry diagram of GRN

Based on previous observations and data presented here, we propose a model for and preliminary gene regulatory network describing *NvashA*-dependent neurogenesis in the early embryonic ectoderm of *Nematostella* (Fig. 9a, b). Based on U0126 phenotypes, we hypothesize that one or more not yet identified kinase signaling cascade(s) acting through MEK provides a global cue that is necessary for neural fates. It is not yet clear if all cells are competent to respond to this cue. However, it appears that the number of competent cells is greater than the number of cells that become neuralized, because evidence suggests that Notch signaling integrates the global cue to restrict a subset of cells to become *Nvath-like-* and *NvsoxB(2)-*positive neural progenitor cells (Fig. 9). Interestingly, we saw no evidence that either *Nvnotch* or *Nvdelta* were affected by U0126 treatment (Additional file 5: Table S2), which suggests that the refining activity of *Nvnotch* is independently controlled. It is not clear which, if either, transcription factor [*Nvath-like* or *NvsoxB(2)*] is nearer the top of the neural cascade. *NvsoxB(2)* is expressed maternally (Additional file 2: Figure S1), and both genes appear to be upregulated at approximately the same time in normal development (Additional file 2: Figure S1; Additional file 10: Figure S5). However, increased *Nvath-like* expression accumulates before *NvsoxB(2)* increases in animals with inhibited Notch activity [9]. Additionally, *NvsoxB(2)* and *NvashA* co-expression can be observed in post-mitotic cells, whereas *Nvath-like* and *NvashA* double-positive cells are never observed [9]. Post-mitotic neurons do not appear to express *Nvath-like* and they lose *NvsoxB2* expression. Lineage-specific pro-differentiation neural markers such as *NvashA* are not expressed until post-mitotic stages*.* The observation that *NvashA* suppresses *Nvath-like* expression suggests that one of its functions is to inhibit neural progenitor identity. This is contrary to reported interactions for *Nvath-like* and *NvashA* [9]. However, the previous study assessed phenotypes at later time points, and thus cannot account for potential phenotypes arising due to sustained loss of a key neurogenic gene causing system-wide defects. Here we look closer at the onset of neurogenesis, which provides less time for potential nonspecific phenotypes to arise. Certainly further efforts are needed to clarify this point. *NvashA* also promotes the expression of distinct individual neuronal subtype markers. The mechanism by which subtype markers are regulated is still unclear, but it is likely that regionally expressed oral–aboral patterning genes and the temporal window in which neural progenitors/neurons are born likely contribute to neural patterning [11, 19, 21].

It is not clear how only a subset of the *NvsoxB(2)* and *Nvath-like* double-positive cells give rise to *NvashA*-expressing cells. *NvsoxB(2)*, *Nvath-like*, and *NvashA* expression do not appear to be restricted to a distinct spatial domain [9, 11, 41]. One hypothesis is that progenitors give rise to different daughter cells with distinct identities, and that *NvashA* defines one such identity. This idea would be consistent with temporal patterning observed for neural progenitor lineages in *Drosophila* [58]. Alternatively, the time and position at which a progenitor is born might determine its identity and subsequently the identity of the neurons it generates [59, 60]. Regardless, functional studies support a much broader role for the progenitor marker *NvsoxB(2)*, and suggest that some additional mechanism is acting to restrict *NvashA* expression to a subset of the *NvsoxB(2)*-positive progenitors.

### Additional putative neural genes identified here

We have identified 18 new genes expressed early (in late blastula/early gastrula) stages that are candidate neural regulators or neural subtype markers. The earliest expressed gene in our temporal analysis was *Nvath-like*, which had been previously identified as an early-acting neurogenic gene. Two genes turned on slightly after *Nvath-like* were *Nvhes3-like* and *NvfoxD3-like*. Previous studies suggest that *Nvhes3-like* is not sufficient to regulate neurogenesis at this stage, but it cannot yet be ruled out as a neural regulator because efficient *Nvhes3-like* knockdowns are not yet reported [36]. The next two genes to be expressed were *Nvsox2* and *Nvfoxq2-like*. Again, the broad expression of *Nvsox2* was similar to that of *NvfoxD3-like* and suggests that it may act broadly to regulate neurogenesis. *Nvfoxq2-like* genes displayed limited expression in the aboral region, suggesting their neurogenic potential is limited to neurons arising from that domain. The late-onset genes displayed both broad and restricted expression patterns, suggesting that at least some of these genes have roles regulating distinct neural subtypes. This is supported by the inclusion of *NvashA* as a late-onset gene. However, it must be noted that neurogenesis in *Nematostella* is a continuous process and late onset alone is not sufficient to suggest that a gene acts at the later steps in neurogenesis. Regardless, we have identified a number of putative neural genes, and future work will allow us to both determine which genes are definitively neural, and where each gene fits into the regulatory networks describing the earliest born neural subtypes in *Nematostella*.

### Neural induction in *Nematostella*

MAPK/MEK activity is required for the expression of key neural progenitor markers, suggesting that it might be a component of neural induction in *Nematostella*. Loss of neurogenesis following treatment with U0126 implies that a positive neurogenic cue is disrupted by U0126 treatment. It is tempting to speculate that FGF signaling may be responsible for neural induction, because FGF is a positive regulator of neural induction during vertebrate development [15] and MEK activity has been described to be downstream of FGF signaling in other systems. However, we report no phenotype when the broadly expressed *NvfgfRa* receptor was knocked down (Fig. 7). However, we still suspect that some MAPK signaling through MEK is necessary for neurogenesis. α–phospho-Erk staining was detected throughout the embryonic ectoderm, and this staining was reduced in U0126-treated animals (Fig. 2). Recently, MAPK signaling was shown to positively promote neurogenesis in salamander animal cap explants, in part by inhibiting BMP responsiveness due to decreased *smad1* expression [13]. However, expression of the putative neural marker *Nvgata* is not sensitive to the level of *Nvbmp2/4* in *Nematostella* gastrula [23]. Thus, the potential mechanism by which MAPK/MEK signaling promotes neural fates is not yet understood in *Nematostella*.

### Possibly multiple neural inductive events during *Nematostella* neurogenesis

Multiple observations raise the hypothesis that distinct mechanisms may be necessary for neurogenesis in different spatiotemporal windows in *Nematostella*. First, *Nvbmp2/4* does not appear to impact neurogenesis at early embryonic stages, but expression of neural markers such as *Nvelav1* and *NvashA* are sensitive to the levels of *Nvbmp2/4* and *Nvbmp5/8* in planulae [22, 23]. Second, *NvashA* was not detected in U0126-treated embryos at early gastrula stages, but animals that were allowed to continue developing in the presence of U0126 begin to show *NvashA* expression, albeit in very few cells (Additional file 12: Figure S7). Third, very few *NvLWamide*-positive and *NvanthoRFamide*-positive cells were able to form in U0126-treated animals, which suggests that a subset of *NvLWamide* and *NvanthoRFamide* neurons may be U0126-independent. The potential for multiple neural programs acting in *Nematostella* suggest that efforts to isolate specific neurons born in unique spatiotemporal windows as well developing conditional alleles to investigate later time points in isolation will improve our understanding of neurogenesis in *Nematostella*.

### Patterning genes identified in the UO126 microarray

In addition to the genes with a neural-like expression pattern, our forward molecular approach identified ~40 genes downstream of MEK that were expressed at the animal pole, including genes expressed in the presumptive endomesoderm and genes expressed at the aboral pole. These data suggest that MAPK acts broadly in the embryo to regulate germ layer specification as well as regional identities associated with axial patterning. UO126 treatment has been reported to suppress formation of the apical tuft at the aboral pole of the larva, likely via inhibition of NvFGFRa-mediated MAPK signaling [24, 26]. However, early embryonic patterning of the aboral domain has been largely understudied, and preliminary data suggest that distinct mechanisms act at early (embryonic) and late (larval) stages of aboral patterning [19]. Additional FGF receptors and ligands are detected in the oral domain of *Nematostella*, suggesting that FGF-mediated MAPK signaling normally regulates U0126 targets identified that display oral/animal pole expression [25]. However, targeted gene-specific knockdowns will be important to further determine exact mechanisms by which MEK activity impacts aboral and/or germ layer specification.

## Conclusions

Our data indicate MAPK signaling is necessary for neurogenesis in the embryonic ectoderm of *Nematostella.* Our work also built upon previous observations to improve our understanding of the molecular mechanisms underlying cnidarian neurogenesis and described two transgenes that describe distinct neural subtypes in *Nematostella.* Lastly, we identified ~120 signaling molecules and transcription factors that act downstream of MEK in MAPK signaling. Future characterization of the genes will provide critical cues about the early patterning mechanisms acting during *Nematostella* development, which will be important to allow improved understanding about the origin and evolution of neurogenesis, axial patterning, and endomesoderm specification.

## Methods

### Culture and spawning of *Nematostella vectensis*

Adult *Nematostella* were cultivated either at the Kewalo Marine Laboratory/PBRC of the University of Hawaii (USA), the Whitney Laboratory for Marine Bioscience of the University of Florida (USA), the Institute for Research on Cancer and Aging of the University of Nice-Sophia-Antipolis (FRA), or Lehigh University (USA) according to the protocol described in [45]. Males and females were kept in separate glass bowls (250 ml) in 1/3× seawater (salinity: 12 pp) [5] at 17 °C in the dark and water was changed weekly. Animals were fed three/four times a week with pieces of oysters or brine shrimps. Manipulating the light cycle induced spawning and oocytes and sperm were collected separately [4]. The gelatinous mass around the eggs was removed with 4 % L-Cystein in 1/3× seawater before fertilization and then washed three times with 1/3× seawater. For the simultaneous development of the embryos, all oocytes were fertilized in glass dishes at the same time with 0.5 ml of diluted sperm. Fertilized eggs were kept in dark in filtered 1/3× seawater at 17 °C until the desired stage.

### U0126 and SU5402 treatments

The MEK (U0126, Sigma, # U120) and FGF (SU5402, Sigma, # SML0443) antagonists were dissolved at a stock concentration of 10 mM in DMSO and added at to 1/3× filtered seawater to generate final concentrations as indicated. For SU5402, analysis was done at a final concentration of 20 μM, and for U0126 most experiments were conducted at a final concentration of 15 μM. Unless indicated, embryos were treated with the drug directly after fertilization and kept in the dark at 17 °C. If needed, U0126 was replaced every 24 h with fresh solutions to maintain activity. Treatments were compared to DMSO-treated control embryos. Embryos were fixed for in situ hybridization and morphological analysis at indicated stages. mRNA of embryos was extracted 24 hpf (late blastula stage) from two distinct biological replicates for microarray analysis.

### RNA extraction, quantitative PCR, and microarray analysis

RNA extraction, qPCR, and microarray analysis were performed following protocols described in [46]. RNA for qPCR and microarray analysis was isolated with TriPure (Roche, #11667157001) or TRIzol (Invitrogen, #15596-026) according to the manufacturer's instructions, and genomic contamination removed using RNase-free DNase (Qiagen, #79254) for 15 min at 37 °C. The total amount of RNA was quantified with a NanoDrop 2000 spectrophotometer (Thermo Scientific) and the quality analyzed with a Bioanalyzer 2100 (Agilent Technologies Inc.). To generate cDNA, 1 μg of total RNA was used with the Advantage RT-PCR kit (Clontech, #639506) for qPCR analysis. For the fine-scale temporal analysis, total RNA was extracted from the following stages (in hpf): 0, 2, 4, 6, 8, 10, 12, 14, 16, 18, 20, 24, 28, 32, 40, and 48. qPCR analysis using a LightCycler 480 (Roche) utilizing LightCycler 480 SYBR Green 1 Master mix (Roche, #04887352001) was carried out as described previously [11]. Efficiencies for each gene-specific primer pair was determined using a five-fold serial dilution series and only primers with an efficiency ranging from 1.8 to 2.15 were used for further analysis (Additional file 14: Table S6). The housekeeping genes *Nvactin* and/or *Nvgadph* were used to normalize relative fold changes between control and manipulated embryos. Each qPCR analysis was repeated on at least three independent biological replicates and changes were analyzed using a Student’s *t* test. Microarray analysis was conducted by sending 20 μg of total RNA (RIN value >8) to NimbleGen (Iceland) for further cDNA synthesis, labeling, and array hybridization. Two replicates were sent for each control and U0126-treated animals were sent. The 4-plex microarray (72,000 features) is an oligonucleotide-based chip version, custom designed and produced by NimbleGen Systems (Roche). Array data are available from http://www.ebi.ac.uk/arrayexpress/experiments/E-MTAB-4831/. Gene expression levels were normalized in the Nimblescan software according to [47, 48] and fold changes calculated by comparing expression values from control and treated embryos. Array results were screened based on the provided genome annotations assigned to each array spotID. If no clear blast hit or gene information was assigned to the prediction gene model from the Joint Genome Institute, we retrieved the genomic sequences from http://genome.jgi-psf.org/Nemve1/Nemve1.home.html for the given gene and performed manual BLAST (BLASTX) searches [49] against the NCBI database to determine the nature of the predicted gene product. All sequences from genes of interest have been used for BLAST analysis to confirm their nature and to determine previously published genes.

### In situ hybridization, pERK, actin, and nuclear staining

Previously described gene sequences were used to sub-clone into pGemT (Promega, #A3600) from mixed stage cDNA. All other sequences used in this study were isolated in the course of a microarray analysis. Genome predictions as well as expressed sequence tag sequence information were combined to design primers (Additional file 15: Table S7) that allowed the amplification and cloning of genes between 0.5 kb and 2 kb as described above. Accession numbers for all analyzed genes in this study can be found in Additional file 6: Table S3 and Additional file 7: Table S4. Embryo fixation, probe synthesis, and in situ hybridization were performed as previously described [42]. The MegaScript Transcription Kit (Ambion) was used to synthesize 0.5–2 kb digoxigenin (DIG)-labeled (Roche, #11573152910) riboprobes. Hybridization of riboprobes (1 ng/μl) was carried out at 62 °C in 50 % formamide hybe buffer and visualization of the labeled probe was performed using NBT/BCIP as a substrate for the alkaline phosphatase-conjugated anti-DIG antibody (Roche, #11093274910). To analyze embryonic and larval morphology, we used Biodipy FL Phallacidin (Molecular Probes/Invitrogen, #B607) and propidium iodide (Sigma, #81845) to stain f-actin and the cell nuclei respectively as described previously [61]. To analyze embryonic localization of activated ERK, we used a monoclonal antibody that recognizes a phosphorylated epitope of the activated form of ERK [Phospho-p44/42 MAPK (Erk1/2) (Thr202/Tyr204); Cell Signaling, # 4370)]. Antibodies were diluted at 1:200 in blocking solution (PBT (Phosphate Buffered Saline + 0.1 % Tween) + 10 % normal goat serum) overnight at 4 °C. Following six washes in PBT, embryos were incubated with the secondary antibody (anti-rabbit Ig), and diluted at 1:250 for at least 4 h to overnight at 4 °C on a shaking rocker. Phosphate-buffered saline (PBS) was used for washes between antibodies. Specimens were mounted in 80 % glycerol.

### Imaging

In situ hybridization images were taken on either a Zeiss AxioScop 2 mounted with an Axiocam camera triggered by Axiovision software (Carl Zeiss), a Zeiss Axio Imager A2 mounted with a Canon 6D triggered by Canon professional software, or a Nikon NTi using a Nikon DS-Ri2 color camera and the Elements software (Nikon). All expression patterns described here have been submitted to Kahi Kai, a comparative invertebrate gene expression database [62] hosted at http://www.kahikai.org/index.php?content=genes. Scoring of treatment phenotypes was performed on either a Zeiss Z-1 Axio imager or a Zeiss Axio Imager A2 microscope and confocal imaging was conducted on either a Zeiss LSM710 or Zeiss LSM Exciter microscope running the LSM ZEN software (Carl Zeiss). Fluorescent images were false-colored. The fluorescent channels were merged using ImageJ (http://rsbweb.nih.gov/ij/) and cropped to final size in Photoshop Cs6 (Adobe Inc.). Confocal images for Fig. 5 were processed using Imaris 8.1 (Bitplane).

### Microinjection of mRNA and morpholinos

*NvashA:venus* or *venus* mRNA was injected into embryos at 150 ng/μl as previously described [6, 11, 36]. Antisense translation blocking MO against *Nvfgfra* [24] and *NvashA* [11] (GeneTools Inc.; Philomath, OR, USA) and a control MO (5′ AATAAAAAGAATGCCCCCTCACCTCT 3′) with no known targets in the predicted *Nematostella* genome were injected at 1 mM concentrations.

### Transgenic strain generation

To generate transgenic animals we amplified genomic DNA from position Scaffold21: 1349994-1347681 for *Nv239910* (serum amyloid A-like) and from Scaffold60: 1049346-1046951 for *Nv242283* (*NvLWamide-like).* Numbers correspond to genomic positions available in the *Nematostella* genome version 1.0 http://genome.jgi.doe.gov/Nemve1/Nemve1.home.html. We cloned each fragment into the pNvT vector in front of the *mcherry* coding sequence as previously reported [37]. Animals were injected as previously reported [37], and stable F1 lines were identified by screening for fluorescence and outcrossing to wild-type animals.

## Additional files

Additional file 1: Table S1. Dose-dependent effects of U0126 on *Nvsprouty*, *Nvbrachyury*, and *Nv-fgfA1* expression. Dose-dependent effects of U0126 analyzed by in situ hybridization. Analyzed U0126 concentration as indicated in Row 1 and number of embryos with phenotype scored based on expansion/reduction of the domain of expression as indicated in the column on the right. (XLS 33 kb) Additional file 2: Figure S1. Summary of *NvsoxB(2)* RNA-seq data. Plot used data obtained from [33] from two duplicate RNA-seq data sets that generated transcriptomes over the first 19 hpf. Black trace shows average of two replicates. (JPG 471 kb) Additional file 3: Figure S2. Changes in *NvashA*, *Nvath-like*, and *NvsoxB2* expression following drug treatments or in with increased Notch activity. mRNA in situ hybridization for *NvsoxB(2)* (**A**) and *NvashA* (**B**) in DMSO-treated control animals grown to early planula stages (48 hpf at 22 °C). *NvsoxB(2)* (**A’**) and *NvashA* (**B’**) expression in same stage animal treated with U0126 from 24 to 48 hpf or treated with SU5402 from 24 to 48 hpf (**B”**). mRNA in situ expression of *Nvath-like* (**C**) and *NvsoxB(2)* (**D**) in control embryos injected with the *venus* mRNA. Expression of *Nvath-like* (**C’**) and *NvsoxB(2)* (**D’**) in animals injected with *NvnotchICD*:*venus* (the intracellular domain of the Notch receptor), which has been previously shown to hyperactivate Notch signaling. Embryos were classified and quantified as the percent having normal expression, weak expression, or no expression. The phenotypic class with the highest percentage of embryos is indicated. In C and D, the main figure panels are ectodermal focal planes, and insets show deeper focal planes used to confirm embryonic stage. All images are of lateral views with the oral side to the left. (TIF 34682 kb) Additional file 4: Figure S3. NvAshA:Venus localization in U0126-treated animals. NvAshA:Venus protein was detected at high levels and with strong nuclear localization in U0126-treated animals. (TIF 16187 kb) Additional file 5: Table S2. Array data for *NvsoxB(2)*, *Nvnotch*, *Nvdelta*, and neural genes used for transgenes or controls in qPCR experiments. (XLSX 38 kb) Additional file 6: Table S3. Genes with at least a 2-fold change after U0126 treatments based on our array analysis. (XLSX 3983 kb) Additional file 7: Table S4. Selection of 22 genes upregulated after U0126 treatment. Selected genes that were significantly (*P* < 0.05) at least 2-fold upregulated by U0126 treatments. SpotID was the genome protein model ID (JGI) used for the array design. The gene name is based on the best BLAST hit and if available the previously published name(s) is used. Abbreviations are indicated in the table legend at the bottom. References are included in the main text. (XLSX 27 kb) Additional file 8: Table S5. Selection of 100 genes downregulated after U0126 treatments. Selected genes that were significantly (*P* < 0.05) at least 2-fold downregulated by U0126 treatments. SpotID was the genome protein model ID (JGI) used for the array design. The gene name is based on the best BLAST hit and if available the previously published name(s) is used. Color code and abbreviations are indicated in the table legend at the bottom. References are included in the main text. (XLSX 37 kb) Additional file 9: Figure S4. Summary of animal hemisphere and aboral expression genes identified by U0126 array. mRNA in situ patterns are included in a manuscript currently in preparation. (PDF 39 kb) Additional file 10: Figure S5. Gene expression analyzed by quantitative polymerase chain reaction. Salt-and-pepper expressed genes as represented in Fig. 6. High-density gene expression profiles are represented by charts for all genes expressed at the blastula stage [24 hours post fertilization (*hpf*)] and/or gastrula stage (48 hpf) analyzed in this study. The y-axis indicates the relative fold change compared to unfertilized eggs. The *x*-axis indicates developmental time in hpf. Gene names as indicated in the top left corner and the Cp value in unfertilized eggs is indicated in the top right corner of each panel and was used to determine the presence of maternal transcripts in Fig. 6 (Cp > 34.00). *Cp* corresponds to the crossing point, also known as the cycle threshold (*Ct*) value. (PDF 599 kb) Additional file 11: Figure S6. Summary temporal gene expression analysis. Summarized results of the temporal high density profiling (qPCR) used to determine the presence of maternal transcripts and significant zygotic upregulation of a given gene expressed in individual cells of the ectoderm. *n.d.* Not determined. *Genes that have been identified and their spatial blastula and gastrula expression patterns characterized elsewhere (see Additional file 6: Table S3, Additional file 7: Table S4 and Additional file 8: Table S5). (PDF 62 kb) Additional file 12: Figure S7.
*NvashA* expression in animals with varied regiments of U0126 treatment. (**A**) *NvashA* expression in control animals, or in animals treated with U0126 continuously for 48 hours, or from 24 to 48 hpf. Unlike early stages when no *NvashA* expression could be detected (Fig. 3), *NvashA* expression was ultimately detected in U0126-treated animals by 48 hpf. Treatment with U0126 from 24 to 48 hpf reduced *NvashA* expression, but *NvashA* could be detected in many cells, albeit at reduced levels. (**B**) Levels of *NvashA* and *Nvfgfa1* as detected by qPCR at late gastrula stage (48 hpf at 17 °C) in animals injected with the *Nvfgfra* MO or treated with U0126 or SU5402 from 24 to 48 hpf. Relative expression levels are compared to control MO- or DMSO-treated animals respectively. The red box defines 1.5 to −1.5 fold change region. Error bars are standard error. (TIF 11166 kb) Additional file 13: Figure S8.
*NvashA* regulation of target genes in the embryonic ectoderm. Gene expression in *NvashA* morphants (**A–C**), control morpholino (**D–F**), and *NvashA* mRNA injected (**G**, **H**). Quantification below each image represents percent of embryos in each phenotypic class (see key in figure). All images except C and F are aboral views. C and F are oral views. Embryos were classified and quantified as the percent having normal expression, weak expression, or no expression.. The phenotypic class with the highest percentage of embryos is indicated. (TIF 21122 kb) Additional file 14: Table S6. Primer pairs used in this study for qPCR analysis. (XLS 53 kb) Additional file 15: Table S7. Primer pairs used in this study for gene cloning. (XLS 73 kb)
