# Supplementary figures and images for: MAPK signaling is necessary for neurogenesis in Nematostella vectensis
Source: BMC Biol. 2016 Aug 1;14:61. doi: 10.1186/s12915-016-0282-1 (PMC4968017; doi:10.1186/s12915-016-0282-1)

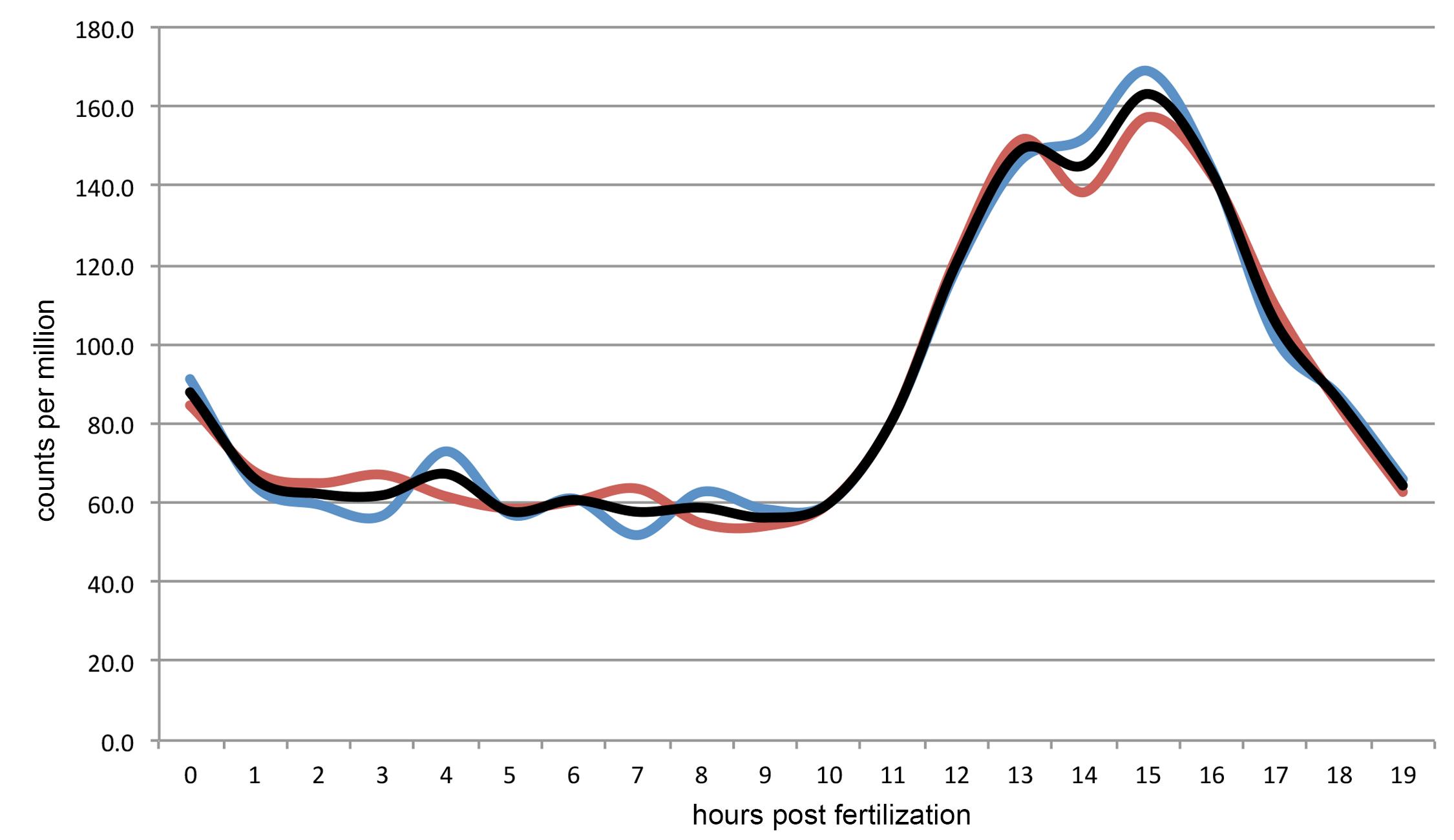

Supplement: Additional file 2: Figure S1. — Summary of NvsoxB(2) RNA-seq data. Plot used data obtained from [33] from two duplicate RNA-seq data sets that generated transcriptomes over the first 19 hpf. Black trace shows average of two replicates. (JPG 471 kb) [file 12915_2016_282_MOESM2_ESM.jpg]

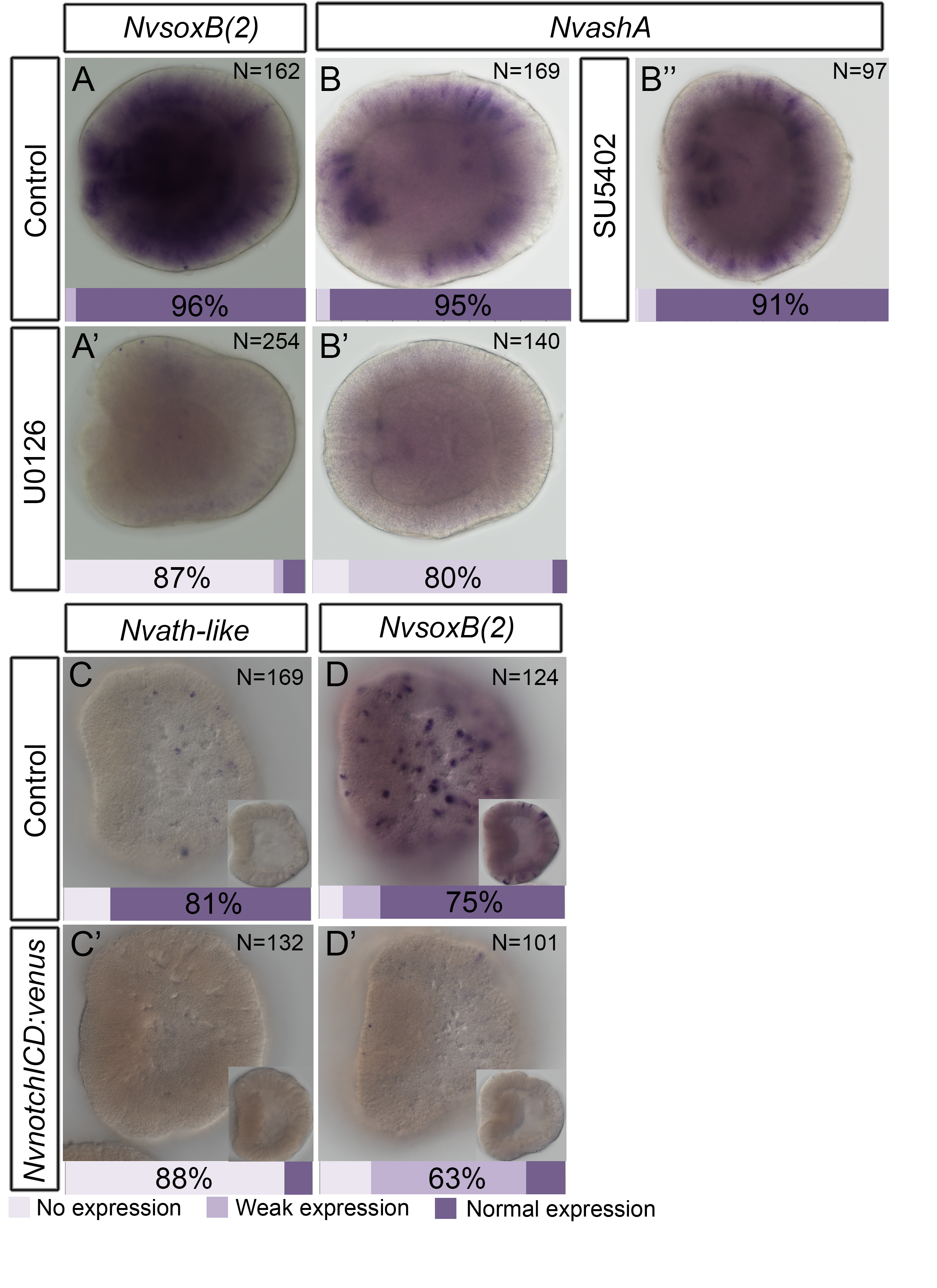

Supplement: Additional file 3: Figure S2. — Changes in NvashA, Nvath-like, and NvsoxB2 expression following drug treatments or in with increased Notch activity. mRNA in situ hybridization for NvsoxB(2) (A) and NvashA (B) in DMSO-treated control animals grown to early planula stages (48 hpf at 22 °C). NvsoxB(2) (A’) and NvashA (B’) expression in same stage animal treated with U0126 from 24 to 48 hpf or treated with SU5402 from 24 to 48 hpf (B”). mRNA in situ expression of Nvath-like (C) and NvsoxB(2) (D) in control embryos injected with the venus mRNA. Expression of Nvath-like (C’) and NvsoxB(2) (D’) in animals injected with NvnotchICD:venus (the intracellular domain of the Notch receptor), which has been previously shown to hyperactivate Notch signaling. Embryos were classified and quantified as the percent having normal expression, weak expression, or no expression. The phenotypic class with the highest percentage of embryos is indicated. In C and D, the main figure panels are ectodermal focal planes, and insets show deeper focal planes used to confirm embryonic stage. All images are of lateral views with the oral side to the left. (TIF 34682 kb) [file 12915_2016_282_MOESM3_ESM.tif]

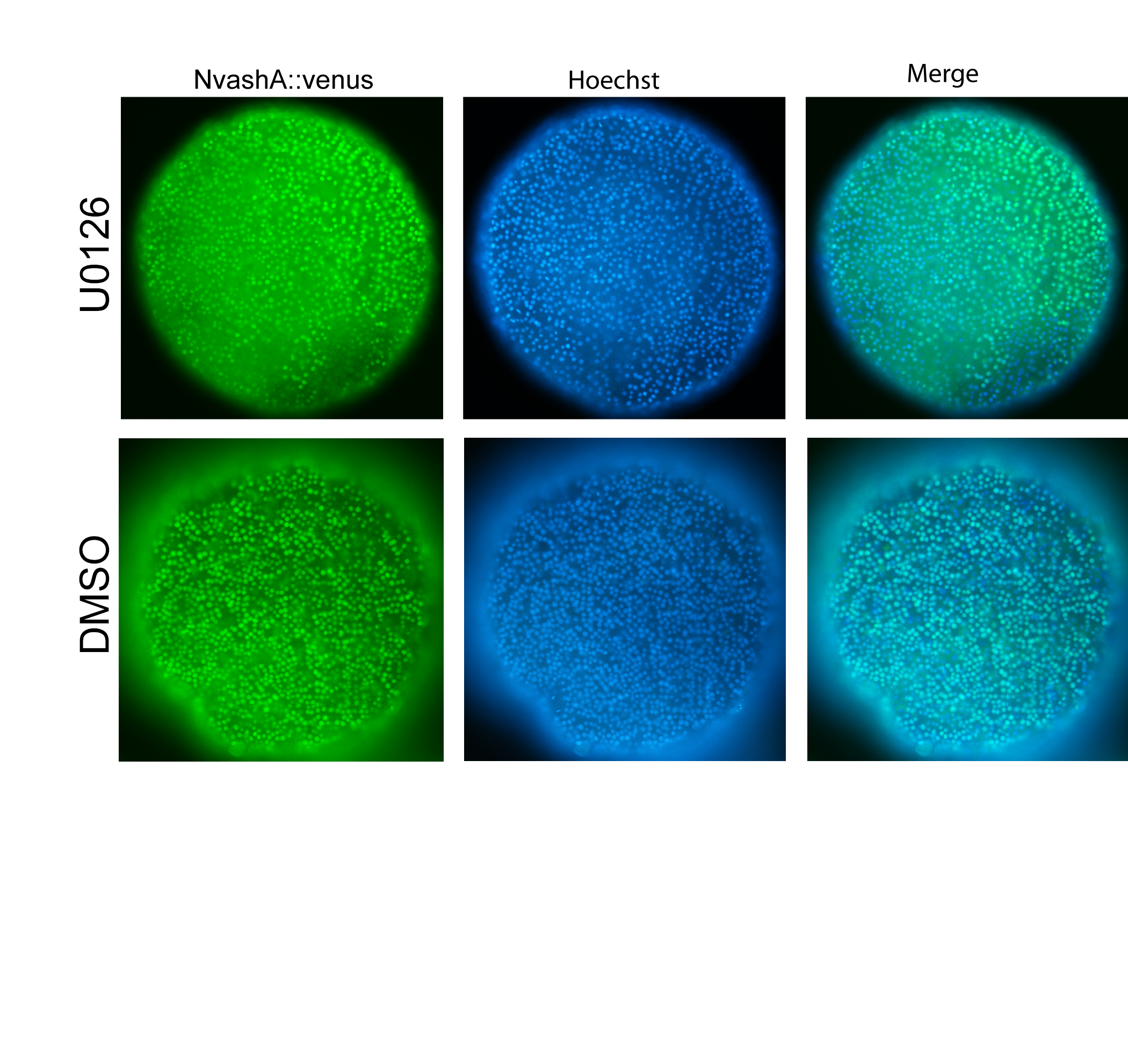

Supplement: Additional file 4: Figure S3. — NvAshA:Venus localization in U0126-treated animals. NvAshA:Venus protein was detected at high levels and with strong nuclear localization in U0126-treated animals. (TIF 16187 kb) [file 12915_2016_282_MOESM4_ESM.tif]

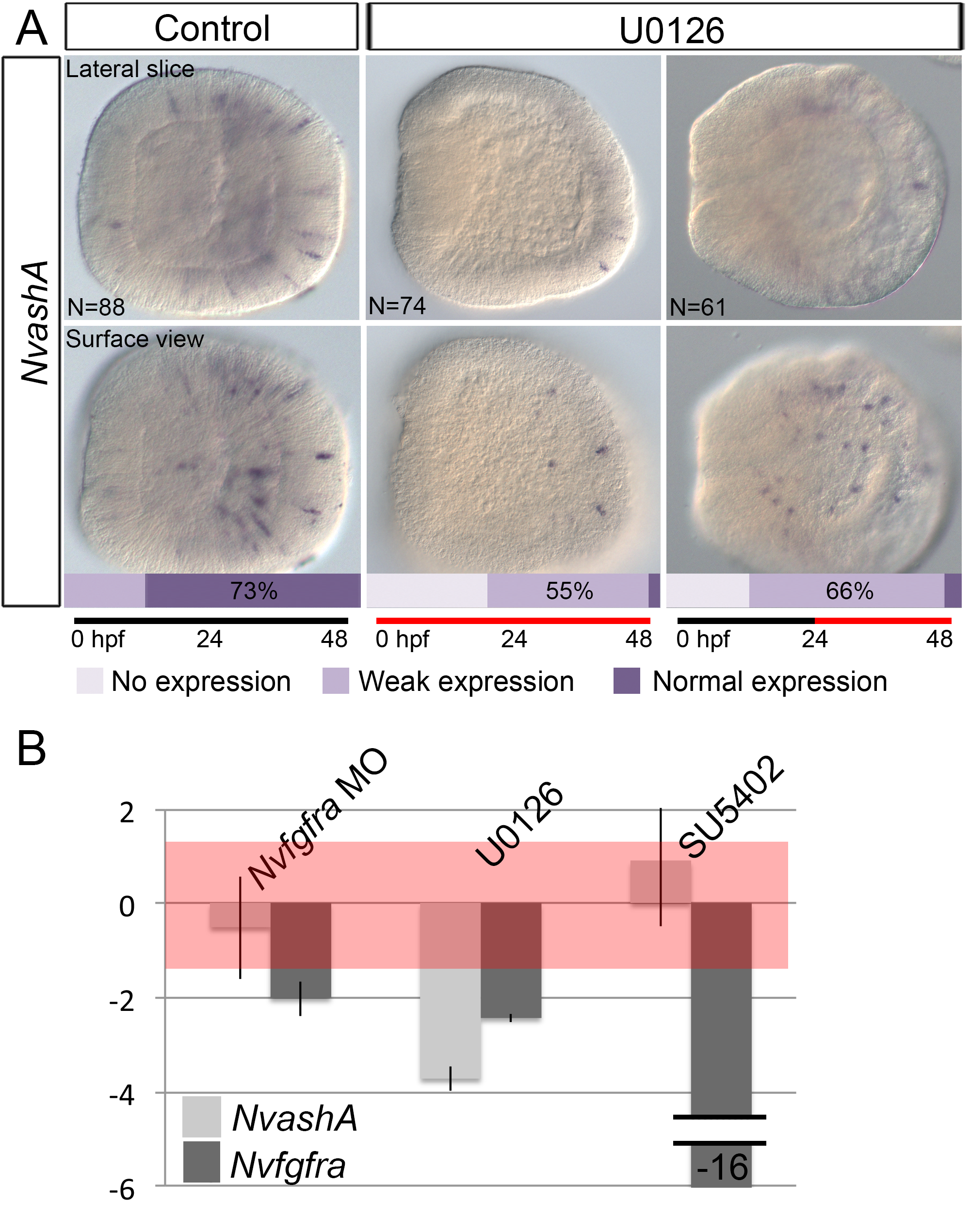

Supplement: Additional file 12: Figure S7. — NvashA expression in animals with varied regiments of U0126 treatment. (A) NvashA expression in control animals, or in animals treated with U0126 continuously for 48 hours, or from 24 to 48 hpf. Unlike early stages when no NvashA expression could be detected (Fig. 3), NvashA expression was ultimately detected in U0126-treated animals by 48 hpf. Treatment with U0126 from 24 to 48 hpf reduced NvashA expression, but NvashA could be detected in many cells, albeit at reduced levels. (B) Levels of NvashA and Nvfgfa1 as detected by qPCR at late gastrula stage (48 hpf at 17 °C) in animals injected with the Nvfgfra MO or treated with U0126 or SU5402 from 24 to 48 hpf. Relative expression levels are compared to control MO- or DMSO-treated animals respectively. The red box defines 1.5 to −1.5 fold change region. Error bars are standard error. (TIF 11166 kb) [file 12915_2016_282_MOESM12_ESM.tif]

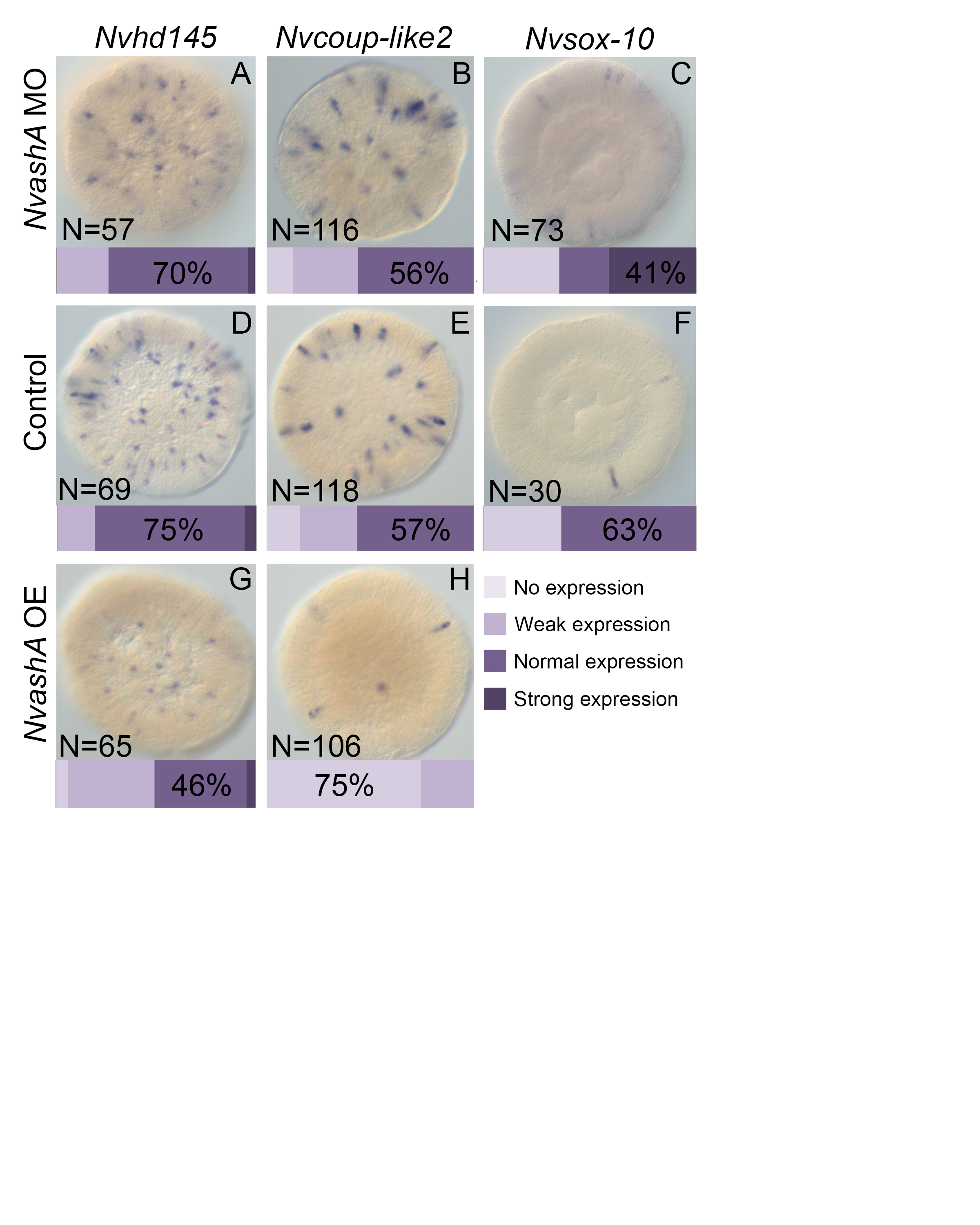

Supplement: Additional file 13: Figure S8. — NvashA regulation of target genes in the embryonic ectoderm. Gene expression in NvashA morphants (A–C), control morpholino (D–F), and NvashA mRNA injected (G, H). Quantification below each image represents percent of embryos in each phenotypic class (see key in figure). All images except C and F are aboral views. C and F are oral views. Embryos were classified and quantified as the percent having normal expression, weak expression, or no expression.. The phenotypic class with the highest percentage of embryos is indicated. (TIF 21122 kb) [file 12915_2016_282_MOESM13_ESM.tif]
