# Supplementary material for: MAPK signaling is necessary for neurogenesis in Nematostella vectensis
Source: BMC Biol. 2016 Aug 1;14:61. doi: 10.1186/s12915-016-0282-1 (PMC4968017; doi:10.1186/s12915-016-0282-1)
Supplement: Additional file 9: Figure S4. — Summary of animal hemisphere and aboral expression genes identified by U0126 array. mRNA in situ patterns are included in a manuscript currently in preparation. (PDF 39 kb) [file 12915_2016_282_MOESM9_ESM.pdf]

Gene	localized expression at 24hpf
fosB-like	animal hemisphere
hes-like2	animal hemisphere
musk-like	animal hemisphere
six4/5	animal hemisphere
eHand-like	animal hemisphere
pdgfr-like	animal hemisphere
mae-like	animal hemisphere
tbx20-like2	animal hemisphere
fz1-like	animal hemisphere
gata	animal hemisphere
kielin-like	animal hemisphere
meis	animal hemisphere
pou-like1	animal hemisphere
pou-like2	animal hemisphere
runt	animal hemisphere
bmp1-like	animal hemisphere
fgfr-like	animal hemisphere
fox1	animal hemisphere
hd058	animal hemisphere
k50-5	animal hemisphere
perlecan-like	animal hemisphere
tbx1	animal hemisphere
ret-like2	animal hemisphere
ephrinB-like	animal hemisphere

Gene	localized expression at 24hpf
lhx6	vegetal hemisphere
sp8/9-like	vegetal hemisphere
fgfA1	vegetal hemisphere
fgfrA	vegetal hemisphere
ax1	vegetal hemisphere
foxq2a	vegetal hemisphere
hmx3-like	vegetal hemisphere
fgfA2	vegetal hemisphere
rx3-like	vegetal hemisphere
sfrp1/5	vegetal hemisphere
dkk124	vegetal hemisphere
hd146	vegetal hemisphere
six3/6	vegetal hemisphere
c-myc-like	vegetal hemisphere
tolloid-like	vegetal hemisphere
foxD1	vegetal hemisphere
wnt7b	vegetal hemisphere
fz5/8	vegetal hemisphere
