# Supplementary material for: MAPK signaling is necessary for neurogenesis in Nematostella vectensis
Source: BMC Biol. 2016 Aug 1;14:61. doi: 10.1186/s12915-016-0282-1 (PMC4968017; doi:10.1186/s12915-016-0282-1)
Supplement: Additional file 10: Figure S5. — Gene expression analyzed by quantitative polymerase chain reaction. Salt-and-pepper expressed genes as represented in Fig. 6. High-density gene expression profiles are represented by charts for all genes expressed at the blastula stage [24 hours post fertilization (hpf)] and/or gastrula stage (48 hpf) analyzed in this study. The y-axis indicates the relative fold change compared to unfertilized eggs. The x-axis indicates developmental time in hpf. Gene names as indicated in the top left corner and the Cp value in unfertilized eggs is indicated in the top right corner of each panel and was used to determine the presence of maternal transcripts in Fig. 6 (Cp > 34.00). Cp corresponds to the crossing point, also known as the cycle threshold (Ct) value. (PDF 599 kb) [file 12915_2016_282_MOESM10_ESM.pdf]

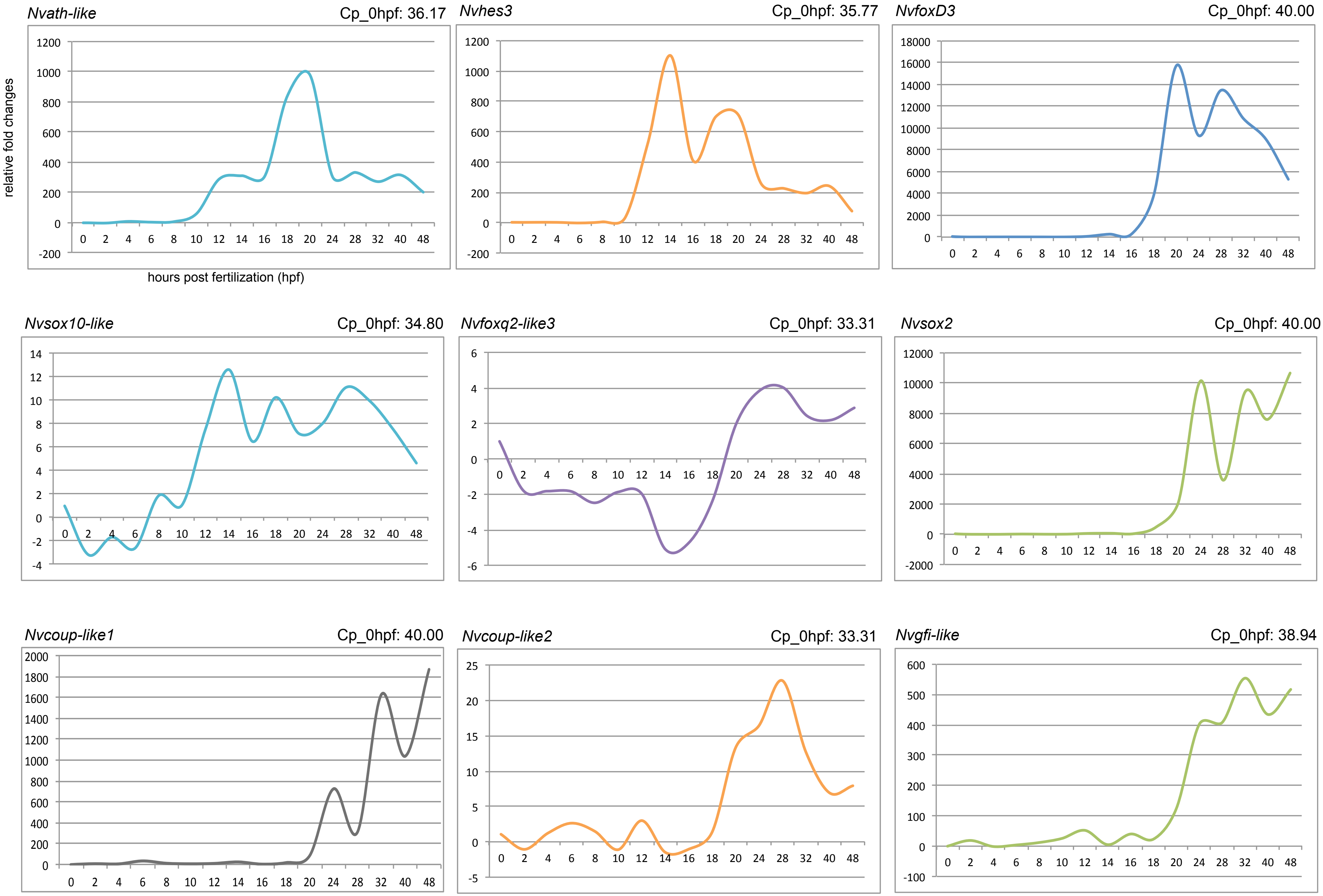
Nvath-like
Cp_0hpf: 36.17
1200
1000
800
600
400
200
0
-200
0 2 4 6 8 10 12 14 16 18 20 24 28 32 40 48
relative fold changes
hours post fertilization (hpf)
Nvhes3
Cp_0hpf: 35.77
1200
1000
800
600
400
200
0
-200
0 2 4 6 8 10 12 14 16 18 20 24 28 32 40 48
NvfoxD3
Cp_0hpf: 40.00
18000
16000
14000
12000
10000
8000
6000
4000
2000
0
0 2 4 6 8 10 12 14 16 18 20 24 28 32 40 48
Nvsox10-like
Cp_0hpf: 34.80
14
12
10
8
6
4
2
0
-2
-4
0 2 4 6 8 10 12 14 16 18 20 24 28 32 40 48
Nvfoxq2-like3
Cp_0hpf: 33.31
6
4
2
0
-2
-4
-6
0 2 4 6 8 10 12 14 16 18 20 24 28 32 40 48
Nvsox2
Cp_0hpf: 40.00
12000
10000
8000
6000
4000
2000
0
-2000
0 2 4 6 8 10 12 14 16 18 20 24 28 32 40 48
Nvcoup-like1
Cp_0hpf: 40.00
2000
1800
1600
1400
1200
1000
800
600
400
200
0
0 2 4 6 8 10 12 14 16 18 20 24 28 32 40 48
Nvcoup-like2
Cp_0hpf: 33.31
25
20
15
10
5
0
-5
0 2 4 6 8 10 12 14 16 18 20 24 28 32 40 48
Nvgfi-like
Cp_0hpf: 38.94
600
500
400
300
200
100
0
-100
0 2 4 6 8 10 12 14 16 18 20 24 28 32 40 48

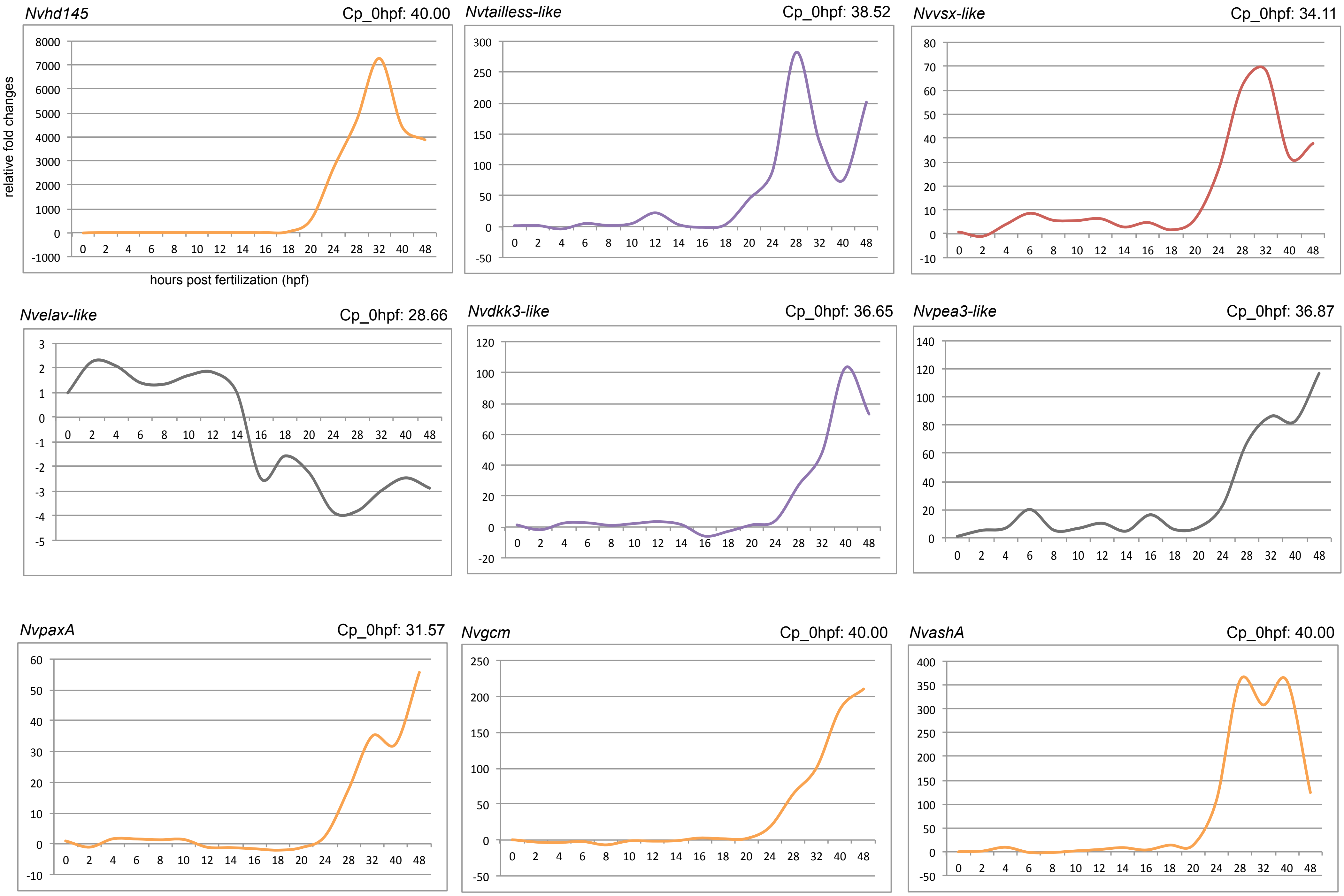

Nvhd145
Cp_0hpf: 40.00
Nvtailless-like
Cp_0hpf: 38.52
Nvvsx-like
Cp_0hpf: 34.11
relative fold changes
hours post fertilization (hpf)
Nvelav-like
Cp_0hpf: 28.66
Nvdkk3-like
Cp_0hpf: 36.65
Nvpea3-like
Cp_0hpf: 36.87
NvpaxA
Cp_0hpf: 31.57
Nvgcm
Cp_0hpf: 40.00
NvashA
Cp_0hpf: 40.00
0 2 4 6 8 10 12 14 16 18 20 24 28 32 40 48

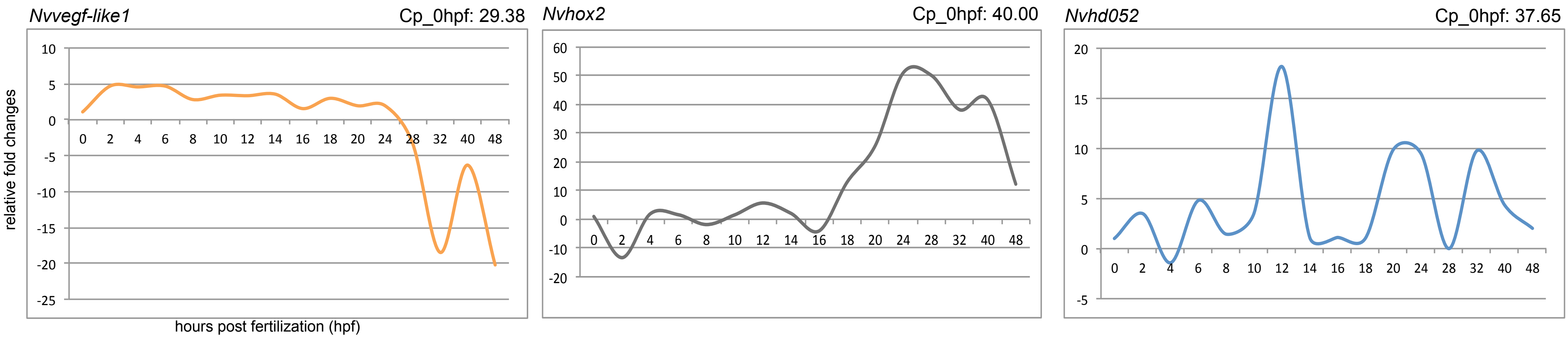

Nvvegf-like1
Cp_0hpf: 29.38
Nvhox2
Cp_0hpf: 40.00
Nvhd052
Cp_0hpf: 37.65
relative fold changes
hours post fertilization (hpf)
0 2 4 6 8 10 12 14 16 18 20 24 28 32 40 48
