# Supplementary material for: MAPK signaling is necessary for neurogenesis in Nematostella vectensis
Source: BMC Biol. 2016 Aug 1;14:61. doi: 10.1186/s12915-016-0282-1 (PMC4968017; doi:10.1186/s12915-016-0282-1)
Supplement: Additional file 11: Figure S6. — Summary temporal gene expression analysis. Summarized results of the temporal high density profiling (qPCR) used to determine the presence of maternal transcripts and significant zygotic upregulation of a given gene expressed in individual cells of the ectoderm. n.d. Not determined. *Genes that have been identified and their spatial blastula and gastrula expression patterns characterized elsewhere (see Additional file 6: Table S3, Additional file 7: Table S4 and Additional file 8: Table S5). (PDF 62 kb) [file 12915_2016_282_MOESM11_ESM.pdf]

Gene	Maternal (Cp)	Zygotic UP	Expression @ 24hpf	Expression @ 48hpf
ath-like	no (36.17)	8-10	★	★
hes3	no (35.02)	10-12	n.d.	★
foxD3-like	no (40.00)	12-14	★	★
sox10-like	no (34.80)	12-14	★	★
foxq2-like3	**yes (33.31)**	16-18	★	★
**hox2**	no (40.00)	16-18	n.d.	★
sox2	no (40.00)	16-18	★	★
coup-like1	no (40.00)	18-20	★	★
coup-like2	**yes (33.31)**	18-20	★	★
gfi-like	no (38.94)	18-20	n.d.	★
hd145	no (40.00)	18-20	★	★
tailless-like	no (38.52)	18-20	n.d.	★
vsx-like	no (34.11)	18-20	★	★
elav-like	**yes (28.66)**	18-20	n.d.	★
dkk3-like3	no (36.65)	20-24	n.d.	★
pea3-like	no (36.87)	20-24	★	★
paxA	**yes (31.57)**	20-24	★	★
gcm	no (40.00)	20-24	★	★
ashA	no (40.00)	20-24	★	★
vegf-like1	**yes (29.38)**	n.d.	n.d.	★
**hd052**	no (37.65)	n.d.	★	★
hes-like3	-	-	★	★
emxLX	-	-	n.d.	★
elav	-	-	★	★
anRFamide	-	-	★	★
soxb(2)	-	-	★	★

ectoderm - salt/pepper expression
